# Canadian Consensus Recommendations on the Management of Extensive-Stage Small-Cell Lung Cancer

**DOI:** 10.3390/curroncol30070465

**Published:** 2023-06-30

**Authors:** Barbara L. Melosky, Natasha B. Leighl, David Dawe, Normand Blais, Paul F. Wheatley-Price, Quincy S.-C. Chu, Rosalyn A. Juergens, Peter M. Ellis, Alexander Sun, Devin Schellenberg, Diana N. Ionescu, Parneet K. Cheema

**Affiliations:** 1 Department of Medical Oncology, BC Cancer-Vancouver Centre, Vancouver, BC V5Z 4E6, Canada; 2 Department of Medicine, Princess Margaret Cancer Centre, University Health Network, University of Toronto, Toronto, ON M5S 1A8, Canada; natasha.leighl@uhn.ca; 3 CancerCare Manitoba Research Institute, CancerCare Manitoba, Department of Internal Medicine, Rady Faculty of Health Sciences, University of Manitoba, Winnipeg, MB R3E 0V9, Canada; ddawe@cancercare.mb.ca; 4 Department of Medicine, Centre Hospitalier de l’Université de Montréal, University of Montreal, Montreal, QC H2X 3E4, Canada; normand.blais.med@ssss.gouv.qc.ca; 5 Department of Medicine, The Ottawa Hospital Research Institute, The Ottawa Hospital, University of Ottawa, Ottawa, ON K1H 8L6, Canada; pwheatleyprice@toh.ca; 6 Division of Medical Oncology, Department of Oncology, Cross Cancer Institute, University of Alberta, Edmonton, AB T6G 1Z2, Canada; quincy.chu@albertahealthservices.ca; 7 Department of Medical Oncology, Juravinski Cancer Centre, McMaster University, Hamilton, ON L8V 5C2, Canada; juergensr@hhsc.ca; 8 Department of Oncology, Juravinski Cancer Centre, McMaster University, Hamilton, ON L8V 5C2, Canada; ellisp@hhsc.ca; 9 Princess Margaret Cancer Centre, Radiation Medicine Program, University Health Network, Toronto, ON M5G 2M9, Canada; alex.sun@rmp.uhn.ca; 10 Department of Radiation Oncology, University of Toronto, Toronto, ON M5G 2M9, Canada; 11 Department of Radiation Oncology, BC Cancer—Surrey Centre, 13750 96 Avenue, Surrey, BC V3V 1Z2, Canada; dschellenberg@bccancer.bc.ca; 12 Department of Pathology, BC Cancer, Vancouver, BC V5Z 4E6, Canada; dionescu@bccancer.bc.ca; 13 Department of Pathology and Laboratory Medicine, University of British Columbia, Vancouver, BC V6T 1Z7, Canada; 14 Division of Medical Oncology, William Osler Health System, University of Toronto, Brampton, ON L6R 3J7, Canada; parneet.cheema@williamoslerhs.ca; 15 Faculty of Medicine, University of Toronto, Toronto, ON M5S 1A8, Canada

**Keywords:** extensive stage small-cell lung cancer, immune checkpoint inhibitors, PD-L1, PD-1, platinum, etoposide, radiation therapy

## Abstract

Small-cell lung cancer (SCLC) is an aggressive, neuroendocrine tumour with high relapse rates, and significant morbidity and mortality. Apart from advances in radiation therapy, progress in the systemic treatment of SCLC had been stagnant for over three decades despite multiple attempts to develop alternative therapeutic options that could improve responses and survival. Recent promising developments in first-line and subsequent therapeutic approaches prompted a Canadian Expert Panel to convene to review evidence, discuss practice patterns, and reach a consensus on the treatment of extensive-stage SCLC (ES-SCLC). The literature search included guidelines, systematic reviews, and randomized controlled trials. Regular meetings were held from September 2022 to March 2023 to discuss the available evidence to propose and agree upon specific recommendations. The panel addressed biomarkers and histological features that distinguish SCLC from non-SCLC and other neuroendocrine tumours. Evidence for initial and subsequent systemic therapies was reviewed with consideration for patient performance status, comorbidities, and the involvement and function of other organs. The resulting consensus recommendations herein will help clarify evidence-based management of ES-SCLC in routine practice, help clinician decision-making, and facilitate the best patient outcomes.

## 1. Introduction

Lung cancer remains the most commonly diagnosed cancer in Canada, with 30,000 estimated new cases in 2022 [1]. Small-cell lung cancer (SCLC) is an aggressive malignancy occurring predominantly in current or former smokers and represents about 15% of all lung cancer diagnoses [2,3]. It is characterized by a high proliferative rate, early development of widespread metastases, and high mortality [2].

Small-cell carcinomas can arise in different organs, including the esophagus, bladder, and cervix; however, over 90% of patients develop it in the lung [4]. Approximately 10% of SCLC presents with mixed histology determined by the presence of features of other non-small-cell carcinoma subtypes [5,6]. However, because there are usually only small tissue biopsies or cytology samples available at diagnosis, the identification of mixed histology is limited at the initial diagnosis and is often detected upon surgical resection or repeat biopsy. Approximately two-thirds of SCLC patients present with metastatic or extensive-stage disease (ES-SCLC) [7]. The most common sites of metastases include lymph nodes, brain, liver, and bones [8]. About 10% of patients present with brain metastases at diagnosis, and 40–50% develop brain metastases during their treatment trajectory [9].

On the other hand, immune checkpoint inhibitors (ICIs; programmed cell death protein 1 (PD-1) and its ligand (PD-L1) and cytotoxic T lymphocyte antigen-4 (CTLA-4)) and targeted therapies have improved the outcomes of numerous malignancies, including non-SCLC (NSCLC), systemic treatments for SCLC remained stagnant for decades despite numerous trials evaluating new therapeutic approaches. It was only recently that several trials demonstrated that adding a PD-L1 inhibitor to etoposide plus platinum (EP) could improve outcomes in patients with ES-SCLC [10,11].

Promising single-arm data with lurbinectedin post-first-line chemotherapy have also expanded options for patients with progressive disease [12].

The emerging evidence, evolving therapeutic landscape, and recent Health Canada approvals and health technology assessment (HTA) funding recommendations will influence practice patterns and the management of ES-SCLC across the country. However, therapeutic advances and regulatory approvals raise questions about how to implement new treatments into current algorithms and how to carefully select patients to enhance response and survival. When attempting to answer these questions, one should keep in mind that patients enrolled in clinical trials often differ from those encountered in routine clinical practice with regards to fitness levels, performance status (PS), and underlying comorbidities. Novel therapies may also present a unique set of toxicities that present new challenges for physicians, especially when selecting treatment for frail patients with poor functional status and/or symptoms related to comorbidities that would not meet clinical trial inclusion criteria. Furthermore, therapeutic approaches and the management of lung cancer in Canada often vary between provinces, territories, and individual centres. The variations depend on funded therapies, as well as access to testing and imaging modalities, ancillary care, and available resources. Thus, therapeutic advances, along with challenges and discrepancies encountered in routine practice, warrant the development of evidence-based consensus recommendations for the management of ES-SCLC. The objectives of the recommendations are to streamline the management of ES-SCLC in Canada while providing clinicians with guidance on how to integrate clinical trial results into routine practice and offer the best available treatment to patients with ES-SCLC.

## 2. Methods

An Expert Panel consisting of medical oncologists, radiation oncologists and a pathologist was convened to develop consensus recommendations (Table 1) for the management of ES-SCLC in Canada based on current clinical evidence and routine practice, as well as existing international guidelines, current Health Canada indications, and HTA drug reimbursement recommendations.

A literature search was conducted within PubMed as well as in oral presentations given at the recent American Association for Cancer Research (AACR), American Society of Clinical Oncology (ASCO), European Society for Medical Oncology (ESMO) and World Conference on Lung Cancer (WCLC) meetings. The search included guidelines, systematic reviews, and randomized controlled trials (RCTs). The relevant literature was selected by the Expert Panel, which convened for several meetings to discuss the available evidence to propose and agree upon specific recommendations.

The strengths of individual recommendations are based on the level of supporting evidence and indicated by the verbs “should”, “could”, and “may”, where “should” indicates the highest level of evidence supported by RCTs; “could” indicates alternative options supported by a lower level of evidence (i.e., non-randomized trials); and “may” indicates expert opinion.

## 3. Pathological Classification and Diagnosis of SCLC

### 3.1. Overview

There are four major classes of lung neuroendocrine tumours (NETs): typical carcinoid, atypical carcinoid, small-cell carcinoma, and large-cell neuroendocrine carcinoma (LCNEC) which are described by the World Health Organization (WHO) [13]. These four entities do not represent a continuum. Although they are biologically related, typical and atypical carcinoids are morphologically and molecularly distinct from the more aggressive tumours (SCLC and LCNEC).

The current sub-classification recognizes two SCLC subtypes: pure SCLC (P-SCLC) and combined SCLC (C-SCLC). C-SCLC is determined by the presence of an NSCLC component, mainly adenocarcinoma, squamous cell carcinoma, or large-cell carcinoma, regardless of the tumour percentage represented by the component. SCLC can co-occur with LCNEC (mixed SCLC) when LCNEC represents at least 10% of the tumour. The presence of any adenocarcinoma component in a C-SCLC or a new SCLC diagnosis in a non-smoker should prompt molecular testing for driver mutations and subsequent consideration of targeted therapy.

Although SCLC has well-established diagnostic criteria, the small tissue samples available from most patients may make the diagnosis of SCLC challenging, especially in cytology specimens. If available, both tissue biopsies and cytology samples should be evaluated and correlated to ensure accurate diagnoses.

SCLC has traditionally been regarded as a morphologic diagnosis. Examination of the tumour cells under light microscopy using routine hematoxylin and eosin (H&E) stained slides was the mainstay of an SCLC diagnosis. Its differentiation from other entities includes lymphoma, basaloid squamous cell carcinoma, and carcinoid tumours. The common cytologic feature of SCLC is the presence of small cells with poorly defined borders, finely granular nuclear chromatin, and absent nucleoli. Other findings include extensive necrosis, prominent apoptotic bodies, and numerous mitoses. While mitotic rates of typical and atypical carcinoids are <2 mitosis/2 mm^2^ and 2–10 mitosis/2 mm^2^, respectively, the mitotic rate in SCLC is usually very high (≥11 mitosis/2 mm^2^).

### 3.2. Immunohistochemistry

The majority of SCLC patients are positive for neuroendocrine markers and confirmatory immunohistochemistry (IHC) has been increasingly used to support the diagnosis of SCLC. The four markers commonly used in clinical practice are synaptophysin, chromogranin A, CD56, and insulinoma-associated protein-1 (INSM-1). Although a subset of SCLC could be negative for all four markers, a diagnosis can still be made morphologically if other entities (e.g., basaloid squamous cell carcinoma and lymphoma) are ruled out by IHC (i.e., p40 and CD45, respectively).

The Ki67 (MIB-1) labeling index is the standard prognostic and predictive biomarker for all gastrointestinal NETs; however, according to the fifth edition of the WHO classification, the Ki67 antigen is not an essential criterion for the diagnosis of lung neuroendocrine tumours. A high Ki67 (>50%, usually 70–100%) is a hallmark of SCLC and LCNEC [14], and its main practical use is in small biopsies with a crush artifact with nuclear features that are difficult to observe [15]. In those situations, a high Ki67 strongly supports the diagnosis of SCLC or LCNEC, while Ki67 less than 20–30% is most likely to be a carcinoid tumour [16]. Ki67 labeling is not used to stratify patients for first-line platinum-based chemotherapy for SCLC [17] nor for radiotherapy [18]. Thyroid transcription factor-1 (TTF-1) is expressed in small-cell carcinoma irrespective of site, and unlike in carcinoids, where TTF-1 can serve as a marker of lung origin, it cannot be used for differentiating SCLC from small-cell carcinoma originating from other sites [19].

PD-L1 expression in SCLC is substantially lower compared to other solid tumours, including NSCLC. Although additional data are needed, evidence from trials that confirmed the efficacy of PD-L1 inhibitors (IMpower133 and CASPIAN) does not support PD-L1 assessment as a predictive biomarker for ICI efficacy [10,11]. Similarly, a high tumour mutational burden (TMB) is not correlated with PD-L1 expression or predictive of treatment outcomes.

### 3.3. Combined Histology Tumours and SCLC Transformation

Several reviews and case reports have discussed the development of C-SCLC [20,21,22]. It is hypothesized that any lung epithelial cell (i.e., neuroendocrine, basal, or totipotent epithelial cell) could differentiate de novo or as the disease progresses under treatment pressure. Approximately 3–10% of NSCLC with epidermal growth factor receptor (EGFR) mutations acquire SCLC morphology and expression of neuroendocrine markers as EGFR-inhibitor resistance evolves [23,24,25]. These tumours are sensitive to standard SCLC treatment [25]. In fact, patients with EGFR-mutated NSCLC and loss of TP53 and retinoblastoma 1 (RB1) are at a higher risk for SCLC transformation [26]. However, even though biallelic loss of function of two tumour-suppressor genes RB1 and TP53 is characteristic of SCLC [13,27,28], the presence of alterations in EGFR, TP53, and RB1 is insufficient to diagnose transformation to SCLC which need to be confirmed with a tissue biopsy indicating histologic features of SCLC).

Unlike carcinoids, SCLC does not occur in the context of multiple endocrine neoplasia type 1 (MEN1) syndrome and does not show somatic or constitutive MEN1 mutations.

### 3.4. Recommendations for Pathological Assessment

1. SCLC should be diagnosed according to the most recent World Health Organization criteria.
2. If available, tissue biopsies and cytology samples should be correlated to ensure the accurate diagnosis of SCLC.
3. The presence of any adenocarcinoma component in a C-SCLC or a new diagnosis of SCLC in a non-smoker should prompt molecular testing for driver mutations for the consideration of targeted therapy.
4. Currently there is no routine predictive biomarker available for SCLC and biomarker testing is not recommended in routine clinical practice.

## 4. Staging

### 4.1. Overview

Historically, a treatment-based staging system introduced by the Veterans Administration Lung Study Group (VALSG) has been used to define the extent of disease in patients with SCLC. LS-SCLC can be defined as a disease that is limited to the ipsilateral hemithorax and regional nodes, which can be included in a single tolerable radiotherapy port. ES-SCLC is a disease beyond the ipsilateral hemithorax and may include diseases exceeding a tolerable radiotherapy port, malignant pleural or pericardial effusion, or hematogenous metastases.

While tumour, node, metastasis (TNM) staging has been the recommended staging approach since the seventh edition of the International Association for the Study of Lung Cancer (IASLC) in 2009 [29], in clinical practice most treatment decisions are still made using VALSG staging [29,30,31]. The simple, two-stage system carries both prognostic importance and implications for treatment. As systemic therapy is recommended for all treatment-eligible patients with tissue diagnosis of SCLC, including those with stage I, the major therapeutic significance of staging is to guide treatment selection.

A complete diagnostic/staging workup should include a physical examination and hematologic and biochemical laboratory profiles. It is important to assess for autoimmune-mediated paraneoplastic neurological symptoms, especially in the context of treatment with PD-1/PD-L1 [32,33,34]. Imaging consists of computed tomography (CT) of the chest, abdomen, and pelvis, and magnetic resonance imaging (MRI) or CT imaging of the brain. MRI of the brain is preferred over CT because it can detect more asymptomatic brain metastases [35]. If there is no evidence of distant metastases on initial scans, then positron emission tomography (PET), where available, or bone scan is reasonable to rule out metastatic disease or to clarify non-specific CT findings. In a review of small prospective studies, 9% of patients were upstaged and 4% were downstaged using PET [36]. However, in jurisdictions with limited access to imaging, waiting may cause an unacceptable delay in treatment. Thus, once a patient has been found to have extensive-stage disease, further imaging is not required, except for brain imaging.

Other tests that may be clinically indicated in selected cases include bone marrow biopsy in the case of cytopenias, lumbar puncture for suspected leptomeningeal disease, and thoracentesis/pericardiocentesis to evaluate pleural/pericardial effusions.

### 4.2. Recommendations for Diagnosis and Staging

5. A complete diagnostic/staging workup should include a physical examination, hematologic and biochemical laboratory profiles, CT chest, abdomen, and pelvis, as well as MRI or CT imaging of the brain.
  - MRI of the brain is preferred over CT to detect asymptomatic brain metastases.
  - PET can be considered when ambiguity exists regarding the diagnosis of LS-SCLC vs. ES-SCLC.
    - PET is only relevant if the disease outside the chest has not been documented.
    - If PET is not available, a bone scan may be used to identify bone metastases.
6. Prompt treatment initiation is of greater importance than complete staging once the extensive disease is demonstrated due to the rapid progression of untreated ES-SCLC. Staging may continue during and immediately after the initiation of treatment.

## 5. First-Line Systemic Therapy

### 5.1. Overview

SCLC is typically highly sensitive to initial chemotherapy and radiotherapy; however, responses are transient, and the vast majority of ES-SCLC patients eventually progress and succumb to recurrent disease (median survival is 10–12 months) [2,37].

Platinum plus etoposide has been the recommended first-line treatment for ES-SCLC for decades. A meta-analysis of individual patient data (32% with LS-SCLC and 68% with ES-SCLC) indicated no difference in response rate (67% vs. 66%) or survival (progression-free survival (PFS; 5.5 vs. 5.3 months), overall survival (OS; 9.6 vs. 9.4 months)) in patients treated with cisplatin- versus carboplatin-containing regimens, suggesting similar efficacy [38]. Thus, toxicity profiles play an important role in decision-making; cisplatin is associated with more non-hematological toxicities (emesis, hearing loss, neuropathy, and nephropathy) [39] and carboplatin with more myelosuppression [40].

Several trials conducted in the early 2000s compared platinum-based and anthracycline-based chemotherapy as first-line options in SCLC. While Sundstrøm et al. demonstrated the survival benefit of platinum-based chemotherapy [41], a phase 3 trial by Baka et al. comparing six cycles of doxorubicin, cyclophosphamide and etoposide (ACE) vs. six cycles of EP found no differences in survival rates at 1-year (34% vs. 38%, *p* = 0.51) and 2-year survival (12% for both arms) [42]. However, the ACE group was associated with a higher risk of neutropenic sepsis (90% vs. 57%, *p* < 0.005). A meta-analysis of 10 RCTs of a cisplatin-containing regimen versus a regimen without platinum demonstrated a significant reduction in risk of death at 6 months (OR 0.87, *p* = 0.03) and 1 year (OR 0.80, *p* = 0.002) with platinum-based regimens [43].

Clinical trials have also evaluated if the substitution of etoposide with a camptothecin analogue, most commonly irinotecan, when given in combination with platinum, can improve survival in ES-SCLC [44,45,46,47,48]. A meta-analysis of 12 RCTs (9 were conducted in East Asia, 2 in multiple countries, and 1 in North America) involving 2030 untreated ES-SCLC patients demonstrated statically significant improvement in 1- and 2-year OS with irinotecan plus cisplatin (IP) compared to etoposide plus cisplatin (risk ratio [RR] 1.16, 95% confidence interval [CI] [1.03–1.31], *p* = 0.02; RR 1.79, 95% CI [1.22–2.61], *p* = 0.003, respectively) [49]. There was no significant difference in objective response rate (ORR) (RR = 1.07, 95% CI [0.99–1.15], *p* = 0.10) or disease control rate (DCR) (RR 1.03, 95% CI [0.96–1.10], *p* = 0.38). Hematologic toxicity, demonstrated by grade 3/4 leukopenia, neutropenia, anemia, and thrombocytopenia was significantly lower with irinotecan plus cisplatin than with etoposide plus cisplatin (all *p* < 0.05). Non-hematologic toxicity indicated by grade 3/4 nausea/vomiting and diarrhea was significantly higher with irinotecan plus cisplatin than with etoposide plus cisplatin (all *p* < 0.05).

Two additional phase 3 RCTs conducted in Germany compared carboplatin plus irinotecan with carboplatin plus etoposide. Results differed, with the first trial showing a slight increase in OS with the irinotecan combination (median survival 8.5 versus 7.1 months; *p* = 0.02) [47], while the second trial survival showed no statistically significant difference (10 vs. 9 months; irinotecan vs. etoposide-based regimens, *p* = 0.06) [50].

### 5.2. The Role of ICIs

Treatment with ICIs has significantly influenced the management of numerous malignancies, including SCLC. The CTLA-4 blocking antibody, ipilimumab, in combination with first-line EP did not improve clinical outcomes when compared to chemotherapy in the phase 3 RCT [51].

IMpower133 [10] and CASPIAN [11], two double-blind, phase 3 RCTs established the role of the PD-L1 inhibitors atezolizumab and durvalumab, respectively, in the treatment of ES-SCLC. IMpower133 assessed adding atezolizumab to carboplatin plus etoposide in 403 patients with previously untreated ES-SCLC [10]. The 1-year OS rates were 51.9% for the atezolizumab plus chemotherapy and 39.0% for placebo plus chemotherapy. At the median follow-up of 22.9 months, a median OS of 12.3 months was seen in the atezolizumab plus chemotherapy arm, while the placebo plus chemotherapy arm had a median OS of 10.3 months (HR, 0.76; 95% CI, 0.60 to 0.95; *p* = 0.0154) [52]. At 24 months, 22% and 17% of patients treated with atezolizumab plus chemotherapy and placebo plus chemotherapy, respectively, were alive. Patients benefited from the addition of atezolizumab, regardless of PD-L1 level on IHC or blood-based TMB status. The rate of grade ≥ 3 adverse events (AEs) was similar in the atezolizumab arm (67.7%) and the chemotherapy alone arm (63.3%).

The CASPIAN trial assessed the efficacy and safety of adding durvalumab ± tremelimumab to etoposide with either carboplatin or cisplatin in 537 patients with previously untreated ES-SCLC [11,53,54]. Most patients (78%) received the carboplatin regimen. The 1-year OS rate was 52.8% for the durvalumab regimen versus 39.3% for chemotherapy alone. After a median follow-up of 25.1 months, the median OS was 12.9 for durvalumab plus chemotherapy versus 10.5 months for placebo plus chemotherapy (HR, 0.71, 95% CI 0.60–0.86; nominal *p* = 0.0003). At 36 months, OS rates were 17.6% and 5.8% for durvalumab plus chemotherapy vs. placebo plus chemotherapy, respectively. The rate of serious AEs was similar in both groups (32% vs. 36%) [54].

The randomized, double-blind, phase 3 KEYNOTE-604 study compared the PD-1 inhibitor pembrolizumab plus EP with placebo plus EP in patients with previously untreated ES-SCLC [55]. Prespecified efficacy boundaries were one-sided (*p* = 0.0048) for PFS and OS (*p* = 0.0128). Four cycles of EP plus pembrolizumab, with a continuation of pembrolizumab for up to 35 cycles, improved PFS (12-month PFS rates of 13.6% with pembrolizumab and 3.1% without pembrolizumab; HR 0.75, 95% CI 0.61–0.91, *p* = 0.0023). The median OS was 10.8 months for pembrolizumab plus chemotherapy versus 9.7 months for chemotherapy plus placebo, with a median follow up of 21.6 months. Although there was a trend to prolonged OS with pembrolizumab plus EP, statistical significance was not reached (HR, 0.80; 95% CI, 0.64 to 0.98; *p* = 0.0164).

The addition of the CTLA-4 inhibitor tremelimumab to durvalumab in the CASPIAN trial did not show additional benefit [11]. Similarly, the phase 3 SKYSCRAPER-02 trial demonstrated that the addition of the anti-TIGIT immunotherapy tiragolumab to atezolizumab plus EP did not provide a benefit over atezolizumab plus EP in patients with untreated ES-SCLC [56]. The median OS with atezolizumab in this study was similar to those seen in the IMpover133 trial and confirmed the benefit of atezolizumab in ES-SCLC.

Both atezolizumab and durvalumab are Health Canada-approved and have positive HTA funding recommendations [57,58,59,60].

In 2018, the PD-1 inhibitor nivolumab was granted accelerated approval by the U.S. Food and Drug Administration (FDA) for the treatment SCLC progressing after platinum-based chemotherapy and at least one other line of therapy. The accelerated approval was based on nivolumab’s effect on surrogate endpoints from the phase I/II CheckMate 032 trial for patients with advanced or metastatic solid tumours [61]. The phase 2 ECOG-ACRIN EA5161 trial (N = 161 ES-SCLC patients with Eastern Cooperative Oncology Group [ECOG] PS 0–1) demonstrated improvement in PFS (HR 0.65 [95% CI, 0.46, 0.91; *p* = 0.012]; median PFS 5.5 vs. 4.6 months) and OS (HR 0.67 [95% CI, 0.46, 0.98; *p* = 0.038]; median OS 11.3 vs. 8.5 months) when nivolumab 360 mg was added to EP for four cycles followed by 240 mg of nivolumab maintenance every 2 weeks, until progression or up to 2 years, compared to EP for 4 cycles followed by observation [62]. However, the subsequent CheckMate 451 phase 3 trial that evaluated the role of nivolumab maintenance failed to provide a statistically significant OS advantage compared to placebo either alone (hazard ratio [HR], 0.84; 95% CI, 0.69–1.02) or in combination with ipilimumab (HR, 0.92; 95% CI, 0.75–1.12) [63] which led to the withdrawal of the nivolumab indication in SCLC from the US market.

#### 5.2.1. Investigational Anti-PD-1/PD-L1 Antibodies

Amongst other investigational therapies are the anti-PD-1/PD-L1 antibodies adebrelimab and serplulimab. Phase 3 trials (ASTRUM-005 with serplulimab [64] and CAPSTONE-1 with adebrelimab [65]) demonstrated significant improvement in OS, Table 2. Based on the ASTRUM-005 data the US FDA granted Orphan Drug Status to serplulimab for SCLC [66]. Currently, these agents are not Health Canada-approved.

#### 5.2.2. What Are the First-Line Treatment Options for Patients with ES-SCLC?

**Figure 1:**
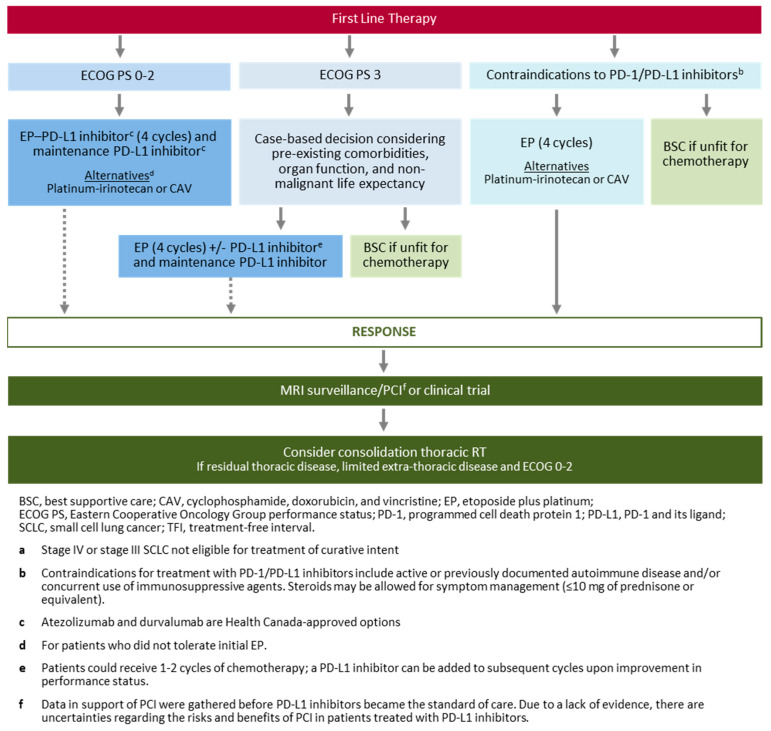
Canadian treatment algorithm for first-line therapy of ES-SCLC ^a^.

When deciding first-line systemic therapy, one should consider the above-described evidence, Health Canada’s indication and access to treatments, as well as patient PS, Figure 1. The CADTH recommendation for funding durvalumab added to platinum and etoposide states that it is reasonable to extrapolate the use of durvalumab to patients with ECOG PS 2. The CADTH clinical expert panel felt that these patients could experience clinical benefit and can experience treatment benefit that leads to better performance status. As our clinical experience indicates the same, we consider some patients with ECOG PS 2 as candidates for treatment with a platinum doublet plus ICIs. For patients with poor PS (ECOG ≥ 3), assessing organ function and whether poor performance is related to the disease or other comorbidities is of particular relevance. As there are no clinical trial data to guide treatment in patients with poor PS, decisions should be made on a case-by-case basis and in consultation with other medical oncologists and/or radiation oncologists. The preferred approach is to offer EP to patients with poor PS due to SCLC rather than comorbidities as there is the potential for a clinically significant response.

Patients with ECOG PS ≥ 3 are often hospitalized and could receive 1–2 cycles of EP to potentially improve their PS, upon which they may receive a PD-L1 inhibitor in subsequent cycles. This approach is mostly driven by physician comfort level, physician experience, and is supported by expert opinion rather than clinical evidence. The lack of supporting clinical evidence may ultimately position this approach outside current funding recommendations in certain provinces.

For PD-L1 ineligible ES-SCLC patients, the preferred first-line treatment is 4–6 cycles of EP. Alternatives could include substituting etoposide with irinotecan in combination with a platinum agent or using a non-platinum-containing regimen such as cyclophosphamide, doxorubicin, and vincristine (CAV); however, these regimens have not been approved in combination with PD-1/PD-L1 inhibitors. It is important to note that irinotecan is a radiosensitizing agent that might interact with radiation therapy. Clinicians should be aware that the tolerability of platinum in combination with irinotecan in this patient population may be challenging and they should monitor for diarrhea and neutropenia to adjust dosing schedules accordingly.

The optimal duration of first-line chemotherapy in ES-SCLC is not well defined; the most common approach is 4–6 cycles. PD-L1 inhibitors are usually continued until disease progression or unacceptable toxicity.

### 5.3. Recommendations for First-Line Systemic Therapy in ES-SCLC

7. Preferred first-line systemic therapy for ES-SCLC should include four cycles of EP in combination with a PD-L1 inhibitor (atezolizumab or durvalumab) if there are no contraindications.
  - The choice between carboplatin and cisplatin should be based on toxicity profile and co-morbidities.
8. Alternatives could include platinum with irinotecan or treating with CAV for a platinum-free regimen. These regimens have not been approved in combination PD-L1/PD-1 inhibitors.
  - If using irinotecan, clinicians should be aware of the increased risk of neutropenia and diarrhea. Irinotecan is a radiosensitizing agent that might interact with radiation therapy.
9. Patients with poor PS (ECOG ≥ 3) may become eligible for a PD-L1 inhibitor if their PS improves after 1–2 cycles of chemotherapy.
  - If available in the treatment jurisdiction, the addition of a PD-L1 inhibitor may be based on clinical judgement and improvement of PS.
10. During systemic therapy and PD-L1 maintenance, patients with ES-SCLC could be assessed with imaging every 2–3 months, depending on disease sites and the burden of the disease.

## 6. Second-Line Therapy and Beyond

### 6.1. Overview

**Figure 2:**
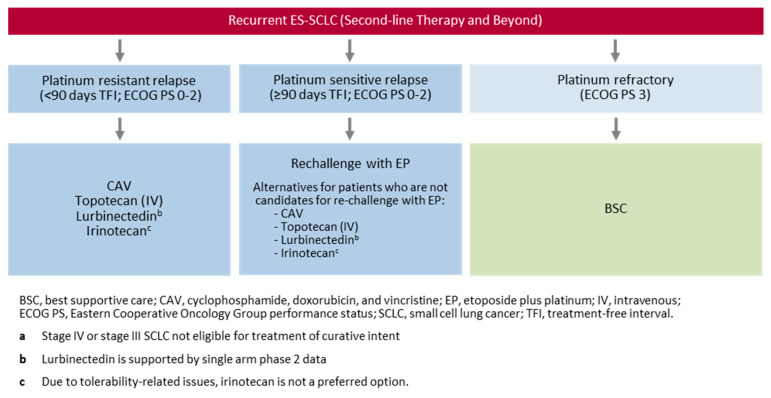
Canadian treatment algorithm for recurrent ES-SCLC ^a^ therapy (second-line and beyond).

Patients who relapse after, or are refractory to, first-line treatment should receive subsequent systemic therapy that provides significant palliation, as shown in Figure 2. However, the likelihood of response depends on the time from initial therapy to relapse [67]; the longer the interval, the higher the likelihood of response to subsequent therapy.

Support for re-challenging with the original platinum regimen over single-agent topotecan in patients who relapse after 90 days or more (defined as platinum-sensitive disease) was confirmed by an open-label, phase 3 RCT conducted in 38 hospitals in France [68]. With a median follow-up of 22.7 months (IQR 20.0–37.3), median PFS was significantly longer with the combination chemotherapy than with topotecan (4.7 months, 90% CI 3.9–5.5 vs. 2.7 months, 2.3–3.2; stratified HR 0.57, 90% CI 0.41–0.73; *p* = 0.0041). OS, however, was not different and suggests that the choice between the treatments may not impact long-term prognosis. In a subset analysis, patients treated with carboplatin and etoposide ≥180 days after initial treatment had greater PFS benefit compared to topotecan (HR 0.23 [95% CI 0.18–0.62]) than patients whose time to progression was between 90 and 180 days (HR 0.70 [0.57–1.05])—reinforcing the relevance of an interval between the treatments.

According to O’Sullivan et al., over half of the ES-SCLC patients in Alberta who initiated second-line treatment were successfully re-challenged with platinum-based chemotherapy [69]. This is consistent with studies conducted in The Netherlands [70] and Sweden [71], while patients in a German study were typically administered topotecan as a second-line therapy [72].

### 6.2. Camptothecins

Topotecan is approved by Health Canada [73] and has been used as second-line therapy for SCLC for several years; however, the funding might vary by province. Beyond topotecan, anthracycline-based regimens are commonly used, including CAV. In an RCT that compared single-agent intravenous (IV) topotecan with CAV as subsequent therapy for patients with SCLC who had relapsed ≥60 days after first-line systemic therapy, both approaches showed similar response rates (topotecan, 24.3%; CAV, 18.3%) and survival (25.0 vs. 24.7 weeks) [74]; however, IV topotecan caused less grade 4 neutropenia (37.8% vs. 51.4%; *p* < 0.001). Compared to CAV, topotecan improved symptoms of dyspnea, anorexia, hoarseness, and fatigue. Both CAV and topotecan are reasonable treatment options and patient preference should be the deciding factor. Dosing schedules for CAV and topotecan differ significantly and can impact both patient preference and the availability of chemotherapy suites—CAV is administered for 1 day every 21 days and topotecan is administered for 5 consecutive days every 21 days.

In a phase 3 RCT that compared the anti-disialoganglioside antibody dinutuximab and irinotecan vs. irinotecan or topotecan for second-line treatment of SCLC in 471 patients, irinotecan administered day 1 every 21 days demonstrated comparable activity to topotecan administered for 5 consecutive days every 21 days [75]. Survival and response rates were not improved for patients receiving dinutuximab/irinotecan versus those receiving irinotecan or topotecan (median OS 6.9 vs. 7.0 vs. 7.4 months [*p* = 0.3132]; median PFS 3.5 vs. 3.0 vs. 3.4 months [*p* = 0.3482].

### 6.3. Lurbinectedin

Lurbinectedin, a selective inhibitor of RNA polymerase II, is approved by Health Canada for the treatment of patients ≥18 years of age with stage III or metastatic SCLC who have progressed on or after platinum-containing therapy [76]. The approval is based on a single-arm, phase 2 trial that included 105 patients with relapsed SCLC [12]. In this trial, single-agent lurbinectedin at a dose of 3.2 mg/m^2^ given every 3 weeks demonstrated promising activity as second-line therapy. The ORR was 35.2% (22.2% in platinum-resistant; 45% in platinum-sensitive patients), and median duration of response (DoR) was 5.3 months. Median OS was 9.3 months (95% CI 6.3–11.8 months). The safety profile was manageable. In December 2022, lurbinectedin received a negative HTA funding recommendation given the ongoing phase 3 trial [77].

A phase 3 RCT (ATLANTIS) with lurbinectedin (2.0 mg/m^2^) plus doxorubicin versus the investigator’s choice of CAV or topotecan did not meet the prespecified superiority endpoint of OS (HR = 0.967, *p* = 0.7032) despite providing other benefits [78]. New combinations of lurbinectedin with other cytotoxic agents, such as irinotecan and PD-1/PD-L1 inhibitors, are being explored. LAGOON, a phase 3, multicentre, open-label, confirmatory clinical trial is assessing the efficacy of lurbinectedin alone or in combination with irinotecan versus investigator’s choice (topotecan or irinotecan) as control arm for the treatment of patients with relapsed SCLC (NCT05153239).

### 6.4. Later-Line Options

For patients progressing on second-line therapy, subsequent options depend on what the patient previously received. CAV might be a reasonable option, if not given previously.

A number of agents, including taxanes, gemcitabine, and temozolomide, have shown activity in patients with relapsed SCLC. Evidence for these treatments comes from small, single-arm phase II trials conducted ≥10 years ago [79,80,81,82].

For patients who are intolerant or have progressed on lurbinectedin and topotecan, the choice of agents depends on the side-effect profile of the agent and patient and provider preferences.

#### What Are the Second- and Later-Line Treatment Options for Patients with ES-SCLC?

From RCT evidence and Health Canada approvals, second-line options for relapsed/refractory ES-SCLC include re-challenge with the initial chemotherapy, CAV, lurbinectedin, irinotecan, and topotecan (Table 3). The current standard of care for chemotherapy-sensitive patients (relapse after ≥90 days of initial treatment) is re-challenge with initial chemotherapy. Options for chemotherapy-resistant ES-SCLC (relapse in <90 days of initial treatment) are CAV, irinotecan, lurbinectedin, topotecan. Due to tolerability-related issues, irinotecan is not a preferred option.

Subsequent treatment may be offered to patients with adequate PS on progression after two lines of therapy. Treatment decisions should be based on the discretion of the treating clinician and patient preference. Subsequent treatment is generally less effective; however, it may provide significant palliation for some patients.

### 6.5. Recommendations for Subsequent Lines of Therapy

11. Retreatment with the initial platinum-based doublet chemotherapy should be considered for platinum-sensitive patients (treatment-free interval ≥ 90 days) who are able to tolerate it.
12. For patients with ES-SCLC who experience progression on or within 3 months of completing first-line chemotherapy, one should consider CAV or IV topotecan. Lurbinectedin could also be considered. Irinotecan is an option.
13. For patients with poor PS (ECOG ≥ 3) with progression while on or after initial therapy, symptom management with best supportive care should be considered.
14. Upon the second progression, subsequent lines of therapy are generally less effective than the initial treatment but may provide significant palliation for some patients. Symptom control and improved quality of life are the primary goals of treatment.

### 6.6. Emerging Options for Relapsed/Refractory ES-SCLC

Emerging options for ES-SCLC, amongst others, include poly adenosine diphosphate-ribose polymerase-(PARP), vascular endothelial growth factor-(VEGF), and delta-like ligand 3-(DLL3) targeting strategies. Several trials suggested the benefit of PARP inhibition when combined with low-dose temozolomide in SCLC [83,84,85] or with the VEGF inhibitor cediranib [86]. Other VEGF inhibitors in clinical development include anlotinib [87] and apatinib [88]. Although the phase III MERU trial failed to show a survival benefit with rovalpituzumab tesirine (an antibody–drug conjugate targeting DLL3) as maintenance therapy [89]. Following first-line platinum-based chemotherapy, other DLL3 targeting strategies are being investigated. A bispecific DLL3/CD3 IgG-like T-cell engaging antibody that potently redirects T-cells to specifically lyse SCLC cells expressing DLL3 is showing promising activity in the early stages of clinical development [90,91].

## 7. Radiation Therapy (RT)

### 7.1. Overview

Both thoracic RT and prophylactic cranial irradiation (PCI) are used to treat in ES-SCLC; however, there are no clinical trials indicating the optimal sequencing of the approaches.

### 7.2. Thoracic RT in ES-SCLC

The role of thoracic RT in ES-SCLC was assessed in three RCTs [92,93,94] and was confirmed in two meta-analyses [95,96]. The studies showed that consolidation with thoracic RT is well-tolerated and lead to fewer symptomatic chest recurrences and improved long-term survival in some patients.

In a trial conducted by Jeremic et al., significant OS benefit was seen when high-dose thoracic RT (54 Gy in 36 fractions over 18 treatment days) was given in combination with chemotherapy after responding to three cycles (median OS was 17 vs. 11 months and 5-year survival was 9.1 vs. 3.7%; *p* = 0.041) [92]. All patients with a complete response in distant metastases had received PCI and acute high-grade toxicity was higher withRT. The inclusion of a highly selective patient population and the high-dose radiation regimen for patients with metastatic disease were likely the reasons that the approach did not become a standard of care.

In 2015, the Chest Radiotherapy Extensive-Stage Trial (CREST) showed a survival benefit for thoracic RT consolidation in patients with ES-SCLC [93]. In CREST, 495 ES-SCLC patients who responded to chemotherapy were randomized to PCI alone or to thoracic RT (30 Gy/10–15 fractions) with PCI. There was no significant improvement in OS or 1-year OS (primary endpoint; 33% vs. 28% for thoracic RT vs. no thoracic RT, (HR 0.84, 95%,CI 0.69–1.01). However, the 2-year OS was 13% vs. 3%, in favour of thoracic RT (*p* = 0.004). Additional analyses showed a statistically significant benefit in OS for thoracic RT in patients with residual intrathoracic disease, and reduction in the risk of disease progression inside the thorax by approximately 50% [97]. No significant toxicity differences were seen between the treatment arms.

Although consolidative thoracic RT was not permitted in the CASPIAN trial with durvalumab [11], a secondary analysis reported that 23 patients treated with durvalumab in combination with EP did receive palliative thoracic RT (3 concurrent and 20 subsequent to study treatment) [98]. There were no reports of additional toxicity, suggesting that palliative doses of thoracic RT could be safely given to patients with residual thoracic disease after completion of chemotherapy with PD-L1 inhibitors. Additional research on thoracic RT and ICIs is needed to establish indications, timing, and dose. A Canadian Consensus recommends consultation with a radiation oncologist to assess the benefits and risks of thoracic RT in patients receiving immunotherapy [99].

#### Recommendations for Thoracic RT

15. RT to the residual primary tumour and lymph nodes could be offered to patients with documented response to systemic therapy presenting with residual thoracic disease, limited extra-thoracic disease and ECOG PS 0–2 who achieve a response after chemotherapy +/− PD-L1 inhibitor.
  - Dosing and fractionation of consolidative thoracic RT should be individualized depending on the symptoms, urgency to treat, and PS.
    - A Total of 30 Gy in 10 fractions was used in the largest randomized trial; 20 Gy in 5 daily fractions may be appropriate due to logistics or for those receiving symptomatic palliation or 40 Gy in 15 daily fractions may be considered in patients with good PS.
    - Consolidative thoracic RT after chemotherapy +/− PD-L1 inhibitor may be considered for patients with documented response to systemic therapy presenting with residual thoracic disease, limited extra-thoracic disease and ECOG PS 0–2 during or before maintenance with a PD-L1 inhibitor.

### 7.3. PCI in ES-SCLC

Several meta and pooled analyses demonstrated that PCI reduces the occurrence of symptomatic brain metastases compared to observation and leads to longer median survival [100,101,102,103].

Two randomized multicentre trials assessed PCI in ES-SCLC [104,105]. In a phase III trial by the European Organization for Research and Treatment of Cancer (EORTC), 286 patients with a response to chemotherapy were randomly assigned to PCI or observation [104]. Radiation doses and schedules varied between institutions and ranged from 20 Gy in five fractions to 30 Gy in 12 fractions. At one year, patients treated with PCI had a significantly decreased incidence of symptomatic brain metastases (15% vs. 40% without PCI, HR 0.27, 95% CI 0.16–0.44). The median OS was increased in patients treated with PCI (6.7 vs. 5.4 months), and one-year OS was significantly increased (27% vs. 13%, HR 0.68 95% CI 0.52–0.88). The risk of extracranial progression did not differ significantly between the two groups (89% vs. 93% at one year); however, a Japanese RCT found that PCI did not improve OS in patients without brain metastases on baseline MRI compared to routine surveillance MRI and treatment of early brain metastases upon detection [105]. In the final analysis, median OS was 11.6 months (95% CI 9.5–13.3) and13.7 months (95% CI 10.2–16.4) in the PCI group and in the observation group, respectively (HR 1.27, 95% CI 0.96–1.68; *p* = 0.094). The cumulative incidences of brain metastases were 15.0% at 6 months, 32.9% at 12 months, and 15.0% at 18 months, in the PCI group and 46.2%, 59.0%, and 63.8%, respectively, in the observation group. Due to the conflicting results summarized above, the Southwest Oncology Group (SWOG) is conducting a phase III study of PCI versus MRI in both LS- and ES-SCLC patients (SWOG S1827, the MAVERICK trial, NCT04155034). American Society for Radiation Oncology (ASTRO) guidelines recommend consultation with a radiation oncologist to assess the benefits and risks of PCI versus MRI surveillance [106].

Increasing age and higher RT doses were found to be the most predictive factors for the development of chronic neurotoxicity. In the Radiation Therapy Oncology Group (RTOG) 0212 trial, 83% of patients >60 years experienced chronic neurotoxicity 12 months after PCI vs. 56% of patients <60 years (*p* = 0.009) [107]. In a large, randomized trial (PCI 99-01), patients receiving a dose of 36 Gy had a higher incidence of chronic neurotoxicity and mortality compared to those treated with 25 Gy [108]. The evidence implies that higher total RT dose (≥36 Gy) should be avoided in patients receiving PCI.

#### 7.3.1. Hippocampal Avoidance (HA) in the Context of PCI

HA has been evaluated in patients with SCLC receiving PCI as a means of reducing neurocognitive decline. Two trials produced conflicting results [109,110] and further data are needed to determine if the approach should become standard practice. Until additional data are available, standard PCI without HA should be considered standard of care and PCI with HA as an acceptable alternative.

#### 7.3.2. Recommendations for PCI

16. For ES-SCLC patients with good PS who have had a complete or very good partial response to chemotherapy +/− PD-L1 inhibitor as part of their first-line systemic therapy, both PCI and observation with regular brain MRI surveillance are acceptable options. An individualized discussion should be held with patients to evaluate the risks and benefits of each approach.
  - The preferred dose for PCI to the whole brain is 25 Gy in 10 daily fractions. A shorter course (e.g., 20 Gy in 5 fractions) may be appropriate in selected patients with ES-SCLC.
  - Higher total RT dose (≥36 Gy) should be avoided in patients receiving PCI.
17. Due to the high risk of developing brain metastases, MRI surveillance imaging (every 3 months during the first year and every 6 months thereafter) for brain metastases should be recommended for all patients regardless of PCI status.

## 8. Treatment of Brain Metastases in ES-SCLC

### 8.1. Overview

About 50% of patients with SCLC develop brain metastases [111,112,113,114] and pose treatment dilemmas due to cognitive changes, diminished blood–brain barrier permeability to systemic therapy, and relatively advanced disease state. Radiotherapy plays a major role in the treatment of brain metastases in patients with SCLC [115].

Brain metastases present at SCLC diagnosis represent a different clinical scenario that requires different management strategies when compared to brain metastases that occur at relapse [111]. The initiation and sequencing of therapies are often based on the severity of thoracic vs. cranial symptoms. Patients with asymptomatic brain metastases revealed at diagnosis or on initiation of systemic therapy are usually initially treated with chemotherapy/chemoimmunotherapy. Pooled data from five studies reported a 66% chemotherapy response rate in 64 patients with brain metastases at diagnosis [116]; however, some data indicate that response rates in the brain are lower than systemic response rates [117] The likelihood of intracranial response may be higher for certain regimens over others (e.g., CAV data are numerically inferior to EP or topotecan for intracranial response) [116,117].

Whole-brain radiotherapy (WBRT) remains an important treatment modality for patients with symptomatic brain metastases. It palliates symptoms, improves intracranial disease control, and reduces the chance of death due to neurologic causes. Unfortunately, the majority of patients experience cognitive deterioration after WBRT [118].

To protect neurocognitive function in patients receiving WBRT, one could consider memantine. In a RCT patients receiving memantine had a longer time before cognitive decline (HR, 0.78; 95% CI, 0.62–0.99, *p* = 0.01) with reduced rates of decline in memory, executive function, and processing speed in patients of any histology receiving WBRT [119].

### 8.2. HA in WBRT

NRG CC001 (NCT02360215), a prospective multi-institutional randomized phase III trial, assessed WBRT plus memantine with or without HA in patients with brain metastases [120]. The risk of cognitive decline was significantly reduced with HA-WBRT plus memantine versus WBRT plus memantine (adjusted HR, 0.74; 95% CI, 0.58 to 0.95; *p* = 0.02). There were no significant differences between treatment arms in OS, intracranial PFS, or toxicity. according to the investigators HA-WBRT plus memantine better preserves cognitive function and as such should be considered a standard of care for patients with good PS receiving WBRT for brain metastases outside the hippocampal region. Recent histology agnostic ASCO-ASTRO guidelines recommend memantine and HA for patients who will receive WBRT, have no hippocampal lesions, and have 4 months or more of expected survival [121].

### 8.3. Stereotactic Radiation Surgery (SRS)

SRS is often selected for different tumour types with small and limited number of brain metastases. Due to the tendency of SCLC to recur intracranially, WBRT is typically selected for patients with SCLC and any degree of intracranial disease. However, WBRT results in greater cognitive decline and because SRS targets lesions and spares normal brain tissue, there is interest in evaluating SRS as an efficacious alternative to WBRT [122,123].

A retrospective multicentre cohort study (FIRE-SCLC) assessed SRS vs. WBRT in 710 patients with SCLC who had a limited number of brain metastases [124]. After controlling for multiple prognostic factors, WBRT was associated with prolonged time to central nervous system progression (HR 0.38, 95% CI 0.26–0.55). However, there was noOS advantage (median OS for WBRT of 5.2 months vs. 6.5 months for SRS; 95% CI 5.5–8.0). On the other hand, in a study with 5952 SCLC patients from the National Cancer Database (200 received SRS, and the rest WBRT), upfront SRS was associated with superior survival (median 10.8 vs. 7.1 months, HR 0.70, 95% CI 0.60–0.81) that persisted on multivariate analyses controlling for comorbidities, extracranial metastases, age, race/ethnicity, and gender [123]. A recent systematic review and meta-analysis found considerable heterogeneity, but outcomes were equitable between SRS and WBRT, concluding that more SRS studies in SCLC are needed [125]. The NRG-CC009 is a phase III trial comparing SRS to HA-WBRT with memantine in patients with ≤10 brain metastases (NCT04804644).

### 8.4. Recommendations for ES-SCLC Patients with Brain Metastases

18. Patients with ES-SCLC who present with asymptomatic brain metastases could receive systemic therapy, followed by radiation therapy upon completion of induction therapy.
19. Those with symptomatic brain metastases should receive radiation therapy, followed by systemic therapy.
20. Serial brain MRI imaging is recommended in patients who have asymptomatic brain metastases and are receiving systemic therapy before brain RT.
  - Brain MRI is recommended every 3 months during the first year and every 6 months thereafter. Brain CT is only an option if MRI cannot be used, because CT is inferior to MRI for detecting brain metastases.
21. In patients with solitary brain metastases, SRS may be an acceptable alternative to WBRT +/− HA, despite the lack of trial data; however, the choice between SRS and WBRT +/− HA depends on the location, size, and number of intracranial lesions and extent of extracranial disease.

## 9. Emerging Biomarkers in SCLC and Future Directions

### 9.1. Overview

Unlike the increasingly personalized clinical approach in NSCLC, where numerous gene alterations and PD-L1 levels guide therapeutic approaches, identifying biomarkers and therapeutic targets in SCLC has been challenging. Recent studies, based primarily on pre-clinical models with SCLC cell lines, genetically engineered mouse models (GEMMs), and patient-derived xenografts (PDXs), have identified distinct subtypes of SCLC defined by different gene expression profiles. In 2018, Huang et al. discovered a non-neuroendocrine, tuft cell variant of SCLC driven by POU2F3 [126]. The discovery prompted the re-evaluation of the SCLC classification in 2019 by Rudin et al. [127] followed by a subsequent classification by Guy at al. in 2021 [128].

Considering evidence gathered from primary human tumours, PDXs, cancer cell lines, and GEMMs, Rudin et al. suggested four major SCLC subtypes. Three of these subtypes were characterized by their expression of distinct transcription factors: high ASCL1 expression (SCLC-A), high NEUROD1 expression (SCLC-N), and high POU2F3 expression (SCLC-P) [127]. The fourth subtype, SCLC-Y (yes-associated protein 1 or YAP1), was found to be associated with poor prognosis, shorter survival, and increased chemoresistance [129]. YAP1 has been proposed to represent one of the subtype-defining markers of SCLC within the “non-neuroendocrine” ASCL1/NEUROD1 double-negative group of tumours associated with decreased insulinoma-associated protein 1 (INSM1) expression and enrichment for intact RB. However, the role of YAP1 as a subtype-defining marker in SCLC requires further study.

Gay et al. employed non-negative matrix factorization followed by consensus clustering [130] to previously published RNAseq data from 81 patients with surgically resected LS-SCLC tumours [131] and 276 treatment-naïve patients with ES-SCLC from the IMpower133 study [10]. The investigators confirmed the previously described NE SCLC phenotypes, SCLC-A and SCLC-N, and identified the novel non-NE “inflamed” subtype (SCLC-I) [128]. SCLC-I tumours lack expression of ASCL1, NEUROD1, and POU2F3 transcription factors; instead, they have a high expression of genes related to immune cell infiltration and immune checkpoints, HLA genes, and interferon-gamma activation. SCLC-I is not specifically characterized by YAP1 expression, differentiating this classification from those proposed elsewhere [127]. This is consistent with subsequent IHC analysis that were not able to identify a separate SCLC-Y population [132]. Interestingly, the distribution of the four subtypes was different in LS-SCLC and ES-SCLC with SCLC-A present in 51% of ES-SCLC and in 36% of LS-SCLC samples. SCLC-N was present in 31% and 23%, SCLC-n in 17%, and 18%, and SCLC-P in 16% and 7% of ES-SCLC and LS-SCLC samples, respectively.

The validation of the four subtypes in cell lines and tumour samples from treatment-naïve metastatic patients demonstrates that their presence is neither stage- nor treatment-specific [128]. The clinical implications of this subtype classification could be relevant for further research because each subtype demonstrates unique vulnerabilities to investigational therapies. Two studies assessed protein expression patterns, tissue distribution and clinicopathological relevance of SCLC molecular subtypes [133,134]. Megyesfalvi Z et al. found non-neuroendocrine subtypes associated with higher OS rates than neuroendocrine subtypes, as well as the correlation between specific subtypes and response to therapy. Dora et al. demonstrated that the innate immune stimulator of interferon genes (STING) expression positively correlates with immune cell infiltration in LS-SCLC. Increased STING-positivity in tumour nests was an independent prognosticator for favourable OS. Although the findings are not ripe for clinical use, they can pave the way for potential biomarker-driven clinical trials that study each SCLC subtype with its matched therapeutic drug.

### 9.2. Recommendation for Emerging Biomarkers

22. Although one can assess novel biomarkers and SCLC subtypes, at present, they should not be used to make treatment-related decisions. Testing for the biomarkers is a research tool that may be used for screening potential trial candidates.

## 10. Conclusions

Systemic and radiation therapy remain the cornerstones of ES-SCLC treatment. Although the magnitude of benefit of adding ICIs to chemotherapy is lower compared to other tumour types, in particular NSCLC, the addition of these agents to ES-SCLC treatment algorithms provided significant improvements in survival outcomes with a known toxicity profile and introduced new multidisciplinary follow-up requirements. Emerging biomarkers and therapeutic targets may further evolve and personalize SCLC treatment approaches. Therapeutic decision-making in routine practice is often impacted by patient lifestyle and comorbidities that, in addition to lung cancer symptom burden, impact fitness level and PS. Although the magnitude of benefit of adding ICIs to chemotherapy is lower compared to other tumour types, in particular NSCLC, the addition of these agents to ES-SCLC treatment algorithms provided significant improvements in survival outcomes with a known toxicity profile and introduced new multidisciplinary follow-up requirements. Real-world evidence can play a significant role in identifying whether studied therapies have a similar impact on patients seen in routine practice, can inform treatment in settings with limited evidence, and can support regulatory approval of new therapies. To that end, the Collaborative Canadian SCLC (CASCADE) database was created to collect data from multiple Canadian sites. The goal is to better understand and characterize approaches to the management of SCLC in Canada through robust RWE that can guide clinical practice [135].

## Figures and Tables

**Table 1:** Summary of Recommendations on the Management of ES-SCLC.

**Recommendations for Pathological Assessment**	
1.	SCLC should be diagnosed according to the most recent World Health Organization criteria.
2.	If available, tissue biopsies and cytology samples should be correlated to ensure accurate diagnosis of SCLC.
3.	The presence of any adenocarcinoma component in a C-SCLC or a new diagnosis of SCLC in a non-smoker should prompt molecular testing for driver mutations for the consideration of targeted therapy.
4.	Currently there is no routine predictive biomarker available for SCLC and biomarker testing is not recommended in routine clinical practice.
**Recommendations for Diagnosis and Staging**	
5.	A complete diagnostic/staging workup should include a physical examination, hematologic and biochemical laboratory profiles, CT chest, abdomen, and pelvis, as well as MRI or CT imaging of the brain. MRI of the brain is preferred over CT to detect asymptomatic brain metastases. PET can be considered when ambiguity exists regarding the diagnosis of limited-stage SCLC (LS-SCLC) vs. ES-SCLC. PET is only relevant if the disease outside the chest has not been documented. If PET is not available, a bone scan may be used to identify bone metastases.
6.	Prompt treatment initiation is of greater importance than complete staging once the extensive disease is evident due to the rapid progression of untreated ES-SCLC. Staging may continue during and immediately after the initiation of treatment.
**Recommendations for First-line Systemic Therapy in ES-SCLC**	
7.	Preferred first-line systemic therapy for ES-SCLC should include four cycles of EP in combination with a PD-L1 inhibitor (atezolizumab or durvalumab) if there are no contraindications. The choice between carboplatin and cisplatin should be based on toxicity profile and co-morbidities.
8.	Alternatives could include platinum with irinotecan or treating with CAV for a platinum-free regimen. These regimens have not been approved in combination with PD-L1/PD-1 inhibitors. If using irinotecan, clinicians should be aware of the increased risk of neutropenia and diarrhea. Irinotecan is a radiosensitizing agent that might interact with radiation therapy.
9.	Patients with poor PS (Eastern Cooperative Oncology Group [ECOG] ≥ 3) may become eligible for a PD-L1 inhibitor if their PS improves after 1–2 cycles of chemotherapy. If available in the treating jurisdiction, the addition of a PD-L1 inhibitor may be based on clinical judgement and improvement of PS.
10.	During systemic therapy and PD-L1 maintenance, patients with ES-SCLC could be assessed with imaging every 2–3 months, depending on disease sites and the burden of the disease.
**Recommendations for Subsequent Lines of Therapy**	
11.	Retreatment with the initial platinum-based doublet chemotherapy should be considered for platinum-sensitive patients (treatment-free interval ≥ 90 days) who are able to tolerate it.
12.	For patients with ES-SCLC who experience progression on or within 3 months of completing first-line chemotherapy, one should consider CAV or IV topotecan. Lurbinectedin could also be considered. Irinotecan is an option.
13.	For patients with poor PS (ECOG ≥ 3) with progression while on or after initial therapy, symptom management with best supportive care should be considered.
14.	Upon the second progression, subsequent lines of therapy are generally less effective than the initial treatment but may provide significant palliation for some patients. Symptom control and improved quality of life are the primary goals of treatment.
**Recommendations for Thoracic Radiation Therapy**	
15.	RT to the residual primary tumour and lymph nodes could be offered to patients with documented response to systemic therapy presenting with residual thoracic disease, limited extra-thoracic disease and ECOG PS 0–2 who achieve a response after chemotherapy +/− PD-L1 inhibitor. Dosing and fractionation of consolidative thoracic RT should be individualized depending on the symptoms, urgency to treat, and PS. In total, 30 Gy in 10 fractions was used in the largest randomized trial; 20 Gy in 5 daily fractions may be appropriate due to logistics or for those receiving symptomatic palliation or 40 Gy in 15 daily fractions may be considered in patients with good PS. Consolidative thoracic RT after chemotherapy +/− PD-L1 inhibitor may be considered for patients with documented response to systemic therapy presenting with residual thoracic disease, limited extra-thoracic disease and ECOG PS 0–2 during or before maintenance with a PD-L1 inhibitor.
**Recommendations for PCI**	
16.	For ES-SCLC patients with good PS who have had a complete or very good partial response to chemotherapy +/− PD-L1 inhibitor as part of their first-line systemic therapy, both PCI and observation with regular brain MRI surveillance are acceptable options. An individualized discussion should be held with patients to evaluate the risks and benefits of each approach. The preferred dose for PCI to the whole brain is 25 Gy in 10 daily fractions. A shorter course (eg, 20 Gy in five fractions) may be appropriate in selected patients with ES-SCLC. A higher total RT dose (≥36 Gy) should be avoided in patients receiving PCI.
17.	Due to the high risk of developing brain metastases, MRI surveillance imaging (every 3 months during the first year and every 6 months thereafter) for brain metastases should be recommended for all patients regardless of PCI status.
**Recommendations for ES-SCLC Patients with Brain Metastases**	
18.	Patients with ES-SCLC who present with asymptomatic brain metastases could receive systemic therapy, followed by radiation therapy upon completion of induction therapy.
19.	Those with symptomatic brain metastases should receive radiation therapy, followed by systemic therapy.
20.	Serial brain MRI imaging is recommended in patients who have asymptomatic brain metastases and are receiving systemic therapy before brain RT Brain MRI is recommended every 3 months during the first year and every 6 months thereafter. Brain CT is only an option if MRI cannot be used, because CT is inferior to MRI for detecting brain metastases.
21.	In patients with solitary brain metastases, SRS may be an acceptable alternative to WBRT +/− HA, despite the lack of trial data; however, the choice between SRS and WBRT +/− HA depends on the location, size, and number of intracranial lesions and extent of extracranial disease.
**Recommendation for Emerging Biomarkers**	
22.	Although one can assess novel biomarkers and SCLC subtypes, at present, they should not be used to make treatment-related decisions. Testing for the biomarkers is a research tool that may be used for screening potential trial candidates.

**Table 2:** Survival Outcomes in Phase 3 RCTs with EP +/− ICI.

Study (Drug)	Trial Design	N Randomization	Median OS (Months) Treatment vs. Control	HR (95% CI)	*p*-Value	Median Follow-Up (Months)	2-Year OS Treatment vs. Control	Health Canada Approved
IMPOWER133 (Atezolizumab)	Double-blind, placebo-controlled	403 1:1 carboplatin and etoposide with atezolizumab or placebo	12.3 vs. 10.3	0.76 (0.60–0.95)	0.0154	22.9	22.0% vs. 16.8%	Yes
CASPIAN (Durvalumab)	Open label	805 1:1 carboplatin or cisplatin and etoposide with durvalumab or placebo	13.0 vs. 10.3	0.73 (0.59–0.91)	0.003	39.4	22.9% vs. 13.9%	Yes
KEYNOTE 604 (Pembrolizumab)	Double-blind, placebo-controlled	453 1:1 carboplatin or cisplatin and etoposide with pembrolizumab or placebo	10.8 vs. 9.7	0.80 (0.64 to 0.98)	0.0164 ^b^	21.6	22.5% vs. 11.2%	No
CheckMate 451 (Nivolumab)	Double-blind	834 1:1:1 nivolumab plus ipilimumab or nivolumab or placebo	9.2 (nivolumab + ipilimumab) vs. 10.4 (nivolumab) vs. 9.6 (placebo)	nivolumab + ipilimumab vs. placebo: 0.92 (0.75 to 1.12) nivolumab vs. placebo: 0.84 (0.69 to 1.02)	Nivolumab + ipilimumab vs. placebo: 0.37	8.9	-	No
ASTRUM-005 (Serplulimab)	Double-blind, placebo-controlled	585 2:1 serplulimab plus chemotherapy or placebo plus chemotherapy	15.4 vs. 10.9	0.63 (0.49–0.82)	<0.001	12.3	-	No
CAPSTONE-1 (Adebrelimab)	Double-blind, placebo-controlled	462 1:1	15.3 vs. 12.8	0.72 (0.58–0.90)	0.0017	13.5	-	No
SKYSCRAPER-02 ^a^ (Tiragolumab)	Double-blind, placebo-controlled	490 1:1 tiragolumab plus atezolizumab plus chemotherapy or placebo plus atezolizumab plus chemotherapy	13.6 (T + A + C) vs. 13.6 (P + A + C)	1.04 (0.79–1.36)	0.7963	14.3	-	Not relevant

^a^ The SKYSCRAPER-02 trial confirms efficacy of atezolizumab seen in the IMPOWER133 trial. ^b^ Not statistically significant. OS, overall survival; T, tiragolumab; A, atezolizumab; C, Carboplatin; P, placebo; RCTs, randomized controlled trials; ICI, immune checkpoint inhibitor; EP, etoposide plus platinum.

**Table 3:** Trials in Support of Second-Line ES-SCLC Treatment Options ^a^.

Trial	Phase	Treatment	ORR	PFS (Months)	OS (Months)
Baize N [68]	3	Carboplatin plus etoposide (re-challenge with chemotherapy)	49%	4.7	7.5
		Topotecan IV	25%	2.7	7.4
Von Pawel J [74]	2	Topotecan IV	24%	3.1	-
		CAV	18%	2.9	-
Edelman MJ [75]	3	Dinutuximab/irinotecan	17.1%	3.5	6.9
		Irinotecan	18.9%	3.0	7.0
		Topotecan IV	20.2%	3.4	7.4
Trigo J [12]	2 (Single arm, open-label) ^b^	Lurbinectedin	34.7%	3.9	9.3
ATLANTIS [78]	3	Lurbinectedin/doxorubicin	31.6%	4	8.6
		CAV or topotecan	29.7%	4	7.6

^a^ Listed in chronological order. ^b^ Led to Health Canada indication.

## Data Availability

The data discussed here are available within the article and its references.

## References

[1] Brenner D.R., Poirier A., Woods R.R., Ellison L.F., Billette J.M., Demers A.A., Zhang S.X., Yao C., Finley C., Fitzgerald N. (2022). Projected estimates of cancer in Canada in 2022. Can. Med. Assoc. J..

[2] Rudin C.M., Brambilla E., Faivre-Finn C., Sage J. (2021). Small-cell lung cancer. Nat. Rev. Dis. Primers.

[3] Bray F., Ferlay J., Soerjomataram I., Siegel R.L., Torre L.A., Jemal A. (2018). Global cancer statistics 2018: GLOBOCAN estimates of incidence and mortality worldwide for 36 cancers in 185 countries. CA Cancer J. Clin..

[4] Berniker A.V., Abdulrahman A.A., Teytelboym O.M., Galindo L.M., Mackey J.E. (2015). Extrapulmonary small-cell carcinoma: Imaging features with radiologic-pathologic correlation. Radiographics.

[5] Travis W., Nicholson S., Hirsch F.R., Pugatch B., Geisinger K., Brambilla E., Gazdar A., Petersen I., Meyerson M., Hanash S.M., Travis W.D., Brambilla E., Müller-Hermelink H.K., Harris C.C. (2004). Small-cell carcinoma. WHO Classification of Tumours of the Lung, Pleura, Thymus and Heart.

[6] Babakoohi S., Fu P., Yang M., Linden P.A., Dowlati A. (2013). Combined SCLC clinical and pathologic characteristics. Clin. Lung Cancer.

[7] Doherty J., Dawe D.E., Pond G.R., Ellis P.M. (2019). The effect of age on referral to an oncologist and receipt of chemotherapy among small-cell lung cancer patients in Ontario, Canada. J. Geriatr. Oncol..

[8] Ko J., Winslow M.M., Sage J. (2021). Mechanisms of small-cell lung cancer metastasis. EMBO Mol. Med..

[9] Quan A.L., Videtic G.M., Suh J.H. (2004). Brain metastases in small-cell lung cancer. Oncology.

[10] Horn L., Mansfield A.S., Szczęsna A., Havel L., Krzakowski M., Hochmair M.J., Huemer F., Losonczy G., Johnson M.L., Nishio M. (2018). First-Line Atezolizumab plus Chemotherapy in Extensive-Stage Small-Cell Lung Cancer. N. Engl. J. Med..

[11] Paz-Ares L., Dvorkin M., Chen Y., Reinmuth N., Hotta K., Trukhin D., Statsenko G., Hochmair M.J., Özgüroğlu M., Ji J.H. (2019). Durvalumab plus platinum-etoposide versus platinum-etoposide in first-line treatment of extensive-stage small-cell lung cancer (CASPIAN): A randomised, controlled, open-label, phase 3 trial. Lancet.

[12] Trigo J., Subbiah V., Besse B., Moreno V., López R., Sala M.A., Peters S., Ponce S., Fernández C., Alfaro V. (2020). Lurbinectedin as second-line treatment for patients with small-cell lung cancer: A single-arm, open-label, phase 2 basket trial. Lancet Oncol..

[13] WHO Classification of Tumours Editorial Board (2021). Thoracic Tumours. WHO Classification of Tumours.

[14] Zheng M. (2016). Classification and Pathology of Lung Cancer. Surg. Oncol. Clin. N. Am..

[15] Pelosi G., Rodriguez J., Viale G., Rosai J. (2005). Typical and atypical pulmonary carcinoid tumor overdiagnosed as small-cell carcinoma on biopsy specimens: A major pitfall in the management of lung cancer patients. Am. J. Surg. Pathol..

[16] Centonze G., Maisonneuve P., Simbolo M., Lagano V., Grillo F., Fabbri A., Prinzi N., Garzone G., Filugelli M., Pardo C. (2023). Lung carcinoid tumours: Histology and Ki-67, the eternal rivalry. Histopathology.

[17] de M Rêgo J.F., de Medeiros R.S.S., Braghiroli M.I., Galvão B., Neto J.E.B., Munhoz R.R., Guerra J., Nonogaki S., Kimura L., Pfiffer T.E. (2017). Expression of ERCC1, Bcl-2, Lin28a, and Ki-67 as biomarkers of response to first-line platinum-based chemotherapy in patients with high-grade extrapulmonary neuroendocrine carcinomas or small-cell lung cancer. Ecancermedicalscience.

[18] Ishibashi N., Maebayashi T., Aizawa T., Sakaguchi M., Nishimaki H., Masuda S. (2017). Correlation between the Ki-67 proliferation index and response to radiation therapy in small-cell lung cancer. Radiat. Oncol..

[19] Agoff S.N., Lamps L.W., Philip A.T., Amin M.B., Schmidt R.A., True L.D., Folpe A.L. (2000). Thyroid transcription factor-1 is expressed in extrapulmonary small-cell carcinomas but not in other extrapulmonary neuroendocrine tumors. Mod. Pathol..

[20] Dong Y., Li Q., Li D., Fang Y., Wang C. (2022). Whole-Process Treatment of Combined Small-cell Lung Cancer Initially Diagnosed as “Lung Squamous Cell Carcinoma”: A Case Report and Review of the Literature. Front. Immunol..

[21] Shen C., Che G. (2022). Case Report: Combined Small-cell Lung Carcinoma with Pulmonary Adenocarcinoma. Front. Surg..

[22] Zhao X., McCutcheon J.N., Kallakury B., Chahine J.J., Pratt D., Raffeld M., Chen Y., Wang C., Giaccone G. (2018). Combined Small-cell Carcinoma of the Lung: Is It a Single Entity?. J. Thorac. Oncol..

[23] Sequist L.V., Waltman B.A., Dias-Santagata D., Digumarthy S., Turke A.B., Fidias P., Bergethon K., Shaw A.T., Gettinger S., Cosper A.K. (2011). Genotypic and histological evolution of lung cancers acquiring resistance to EGFR inhibitors. Sci. Transl. Med..

[24] Yu H.A., Arcila M.E., Rekhtman N., Sima C.S., Zakowski M.F., Pao W., Kris M.G., Miller V.A., Ladanyi M., Riely G.J. (2013). Analysis of tumor specimens at the time of acquired resistance to EGFR-TKI therapy in 155 patients with EGFR-mutant lung cancers. Clin. Cancer Res..

[25] Marcoux N., Gettinger S.N., O’Kane G., Arbour K.C., Neal J.W., Husain H., Evans T.L., Brahmer J.R., Muzikansky A., Bonomi P.D. (2019). EGFR-Mutant Adenocarcinomas That Transform to Small-Cell Lung Cancer and Other Neuroendocrine Carcinomas: Clinical Outcomes. J. Clin. Oncol..

[26] Quintanal-Villalonga A., Taniguchi H., Zhan Y.A., Hasan M.M., Chavan S.S., Meng F., Uddin F., Manoj P., Donoghue M.T., Won H.H. (2021). Multiomic Analysis of Lung Tumors Defines Pathways Activated in Neuroendocrine Transformation. Cancer Discov..

[27] Bunn P.A., Minna J.D., Augustyn A., Gazdar A.F., Ouadah Y., Krasnow M.A., Berns A., Brambilla E., Rekhtman N., Massion P.P. (2016). Small-cell Lung Cancer: Can Recent Advances in Biology and Molecular Biology Be Translated into Improved Outcomes?. J. Thorac. Oncol..

[28] Karachaliou N., Sosa A.E., Rosell R. (2016). Unraveling the genomic complexity of small-cell lung cancer. Transl. Lung Cancer Res..

[29] Vallières E., Shepherd F.A., Crowley J., Van Houtte P., Postmus P.E., Carney D., Chansky K., Shaikh Z., Goldstraw P. (2009). The IASLC Lung Cancer Staging Project: Proposals regarding the relevance of TNM in the pathologic staging of small-cell lung cancer in the forthcoming (seventh) edition of the TNM classification for lung cancer. J. Thorac. Oncol..

[30] Shepherd F.A., Crowley J., Van Houtte P., Postmus P.E., Carney D., Chansky K., Shaikh Z., Goldstraw P. (2007). The International Association for the Study of Lung Cancer lung cancer staging project: Proposals regarding the clinical staging of small-cell lung cancer in the forthcoming (seventh) edition of the tumor, node, metastasis classification for lung cancer. J. Thorac. Oncol..

[31] Pass H., Ball D., Scagliotti G. (2017). IASLC Thoracic Oncology.

[32] Gozzard P., Woodhall M., Chapman C., Nibber A., Waters P., Vincent A., Lang B., Maddison P. (2015). Paraneoplastic neurologic disorders in small-cell lung carcinoma: A prospective study. Neurology.

[33] Raibagkar P., Ho D., Gunturu K.S., Srinivasan J. (2020). Worsening of anti-Hu paraneoplastic neurological syndrome related to anti-PD-1 treatment: Case report and review of literature. J. Neuroimmunol..

[34] Gill A., Perez M.A., Perrone C.M., Bae C.J., Pruitt A.A., Lancaster E. (2019). A case series of PD-1 inhibitor-associated paraneoplastic neurologic syndromes. J. Neuroimmunol..

[35] Seute T., Leffers P., ten Velde G.P., Twijnstra A. (2008). Detection of brain metastases from small-cell lung cancer: Consequences of changing imaging techniques (CT versus MRI). Cancer.

[36] Martucci F., Pascale M., Valli M.C., Pesce G.A., Froesch P., Giovanella L., Richetti A., Treglia G. (2020). Impact of 18F-FDG PET/CT in Staging Patients with Small-cell Lung Cancer: A Systematic Review and Meta-Analysis. Front. Med..

[37] Demedts I.K., Vermaelen K.Y., van Meerbeeck J.P. (2010). Treatment of extensive-stage small-cell lung carcinoma: Current status and future prospects. Eur. Respir. J..

[38] Rossi A., Di Maio M., Chiodini P., Rudd R.M., Okamoto H., Skarlos D.V., Früh M., Qian W., Tamura T., Samantas E. (2012). Carboplatin- or cisplatin-based chemotherapy in first-line treatment of small-cell lung cancer: The COCIS meta-analysis of individual patient data. J. Clin. Oncol..

[39] Hatfield L.A., Huskamp H.A., Lamont E.B. (2016). Survival and Toxicity After Cisplatin Plus Etoposide Versus Carboplatin Plus Etoposide for Extensive-Stage Small-Cell Lung Cancer in Elderly Patients. J. Oncol. Pract..

[40] Bishop J.F., Raghavan D., Stuart-Harris R., Morstyn G., Aroney R., Kefford R., Yuen K., Lee J., Gianoutsos P., Olver I.N. (1987). Carboplatin (CBDCA, JM-8) and VP-16-213 in previously untreated patients with small-cell lung cancer. J. Clin. Oncol..

[41] Sundstrøm S., Bremnes R.M., Kaasa S., Aasebø U., Hatlevoll R., Dahle R., Boye N., Wang M., Vigander T., Vilsvik J. (2002). Cisplatin and etoposide regimen is superior to cyclophosphamide, epirubicin, and vincristine regimen in small-cell lung cancer: Results from a randomized phase III trial with 5 years’ follow-up. J. Clin. Oncol..

[42] Baka S., Califano R., Ferraldeschi R., Aschroft L., Thatcher N., Taylor P., Faivre-Finn C., Blackhall F., Lorigan P. (2008). Phase III randomised trial of doxorubicin-based chemotherapy compared with platinum-based chemotherapy in small-cell lung cancer. Br. J. Cancer.

[43] Pujol J.L., Carestia L., Daurès J.P. (2000). Is there a case for cisplatin in the treatment of small-cell lung cancer? A meta-analysis of randomized trials of a cisplatin-containing regimen versus a regimen without this alkylating agent. Br. J. Cancer.

[44] Noda K., Nishiwaki Y., Kawahara M., Negoro S., Sugiura T., Yokoyama A., Fukuoka M., Mori K., Watanabe K., Tamura T. (2002). Irinotecan plus cisplatin compared with etoposide plus cisplatin for extensive small-cell lung cancer. N. Engl. J. Med..

[45] Hanna N., Bunn P.A., Langer C., Einhorn L., Guthrie T., Beck T., Ansari R., Ellis P., Byrne M., Morrison M. (2006). Randomized phase III trial comparing irinotecan/cisplatin with etoposide/cisplatin in patients with previously untreated extensive-stage disease small-cell lung cancer. J. Clin. Oncol..

[46] Lara P.N., Natale R., Crowley J., Lenz H.J., Redman M.W., Carleton J.E., Jett J., Langer C.J., Kuebler J.P., Dakhil S.R. (2009). Phase III trial of irinotecan/cisplatin compared with etoposide/cisplatin in extensive-stage small-cell lung cancer: Clinical and pharmacogenomic results from SWOG S0124. J. Clin. Oncol..

[47] Hermes A., Bergman B., Bremnes R., Ek L., Fluge S., Sederholm C., Sundstrøm S., Thaning L., Vilsvik J., Aasebø U. (2008). Irinotecan plus carboplatin versus oral etoposide plus carboplatin in extensive small-cell lung cancer: A randomized phase III trial. J. Clin. Oncol..

[48] Zatloukal P., Cardenal F., Szczesna A., Gorbunova V., Moiseyenko V., Zhang X., Cisar L., Soria J.C., Domine M., Thomas M. (2010). A multicenter international randomized phase III study comparing cisplatin in combination with irinotecan or etoposide in previously untreated small-cell lung cancer patients with extensive disease. Ann. Oncol..

[49] Liu Z.L., Wang B., Liu J.Z., Liu W.W. (2018). Irinotecan plus cisplatin compared with etoposide plus cisplatin in patients with previously untreated extensive-stage small-cell lung cancer: A meta-analysis. J. Cancer Res. Ther..

[50] Schmittel A., Sebastian M., Fischer von Weikersthal L., Martus P., Gauler T.C., Kaufmann C., Hortig P., Fischer J.R., Link H., Binder D. (2011). A German multicenter, randomized phase III trial comparing irinotecan-carboplatin with etoposide-carboplatin as first-line therapy for extensive-disease small-cell lung cancer. Ann. Oncol..

[51] Reck M., Luft A., Szczesna A., Havel L., Kim S.W., Akerley W., Pietanza M.C., Wu Y.L., Zielinski C., Thomas M. (2016). Phase III Randomized Trial of Ipilimumab Plus Etoposide and Platinum Versus Placebo Plus Etoposide and Platinum in Extensive-Stage Small-Cell Lung Cancer. J. Clin. Oncol..

[52] Liu S.V., Reck M., Mansfield A.S., Mok T., Scherpereel A., Reinmuth N., Garassino M.C., De Castro Carpeno J., Califano R., Nishio M. (2021). Updated Overall Survival and PD-L1 Subgroup Analysis of Patients with Extensive-Stage Small-Cell Lung Cancer Treated with Atezolizumab, Carboplatin, and Etoposide (IMpower133). J. Clin. Oncol..

[53] Goldman J.W., Dvorkin M., Chen Y., Reinmuth N., Hotta K., Trukhin D., Statsenko G., Hochmair M.J., Özgüroğlu M., Ji J.H. (2021). Durvalumab, with or without tremelimumab, plus platinum-etoposide versus platinum-etoposide alone in first-line treatment of extensive-stage small-cell lung cancer (CASPIAN): Updated results from a randomised, controlled, open-label, phase 3 trial. Lancet Oncol..

[54] Paz-Ares L., Chen Y., Reinmuth N., Hotta K., Trukhin D., Statsenko G., Hochmair M.J., Özgüroğlu M., Ji J.H., Garassino M.C. (2022). Durvalumab, with or without tremelimumab, plus platinum-etoposide in first-line treatment of extensive-stage small-cell lung cancer: 3-year overall survival update from CASPIAN. ESMO Open.

[55] Rudin C.M., Awad M.M., Navarro A., Gottfried M., Peters S., Csőszi T., Cheema P.K., Rodriguez-Abreu D., Wollner M., Yang J.C. (2020). Pembrolizumab or Placebo Plus Etoposide and Platinum as First-Line Therapy for Extensive-Stage Small-Cell Lung Cancer: Randomized, Double-Blind, Phase III KEYNOTE-604 Study. J. Clin. Oncol..

[56] Rudin C.M., Liu S.V., Lu S., Soo R.A., Hong M.H., Lee J.-S., Bryl M., Dumoulin D.W., Rittmeyer A., Chiu C.-H. (2022). SKYSCRAPER-02: Primary results of a phase III, randomized, double-blind, placebo-controlled study of atezolizumab (atezo) + carboplatin + etoposide (CE) with or without tiragolumab (tira) in patients (pts) with untreated extensive-stage small-cell lung cancer (ES-SCLC). J. Clin. Oncol..

[57] Canadian Agency for Drugs and Technologies in Health CADTH Reimbursement Recommendation: Atezolizumab (Tecentriq). https://www.cadth.ca/sites/default/files/DRR/2022/PC0269%20Tecentriq%20for%20NSCLC%20-%20CADTH%20Final%20Recommendation-Final-meta.pdf.

[58] Canadian Agency for Drugs and Technologies in Health CADTH Reimbursement Recommendation: Durvalumab (Imfinzi). https://www.cadth.ca/sites/default/files/DRR/2021/PC0234%20Imfinzi%20-%20CADTH%20Final%20Rec.pdf.

[59] (2023). Atezolizumab (Tecentriq) Product Monograph. Hoffmann-La Roche Limited. Submission Control No: 269664.. https://www.rochecanada.com/PMs/Tecentriq/Tecentriq_PM_CIE.pdf.

[60] (2023). Imfinzi (Durvalumab) Product Monograph. AstraZeneca Canada Inc. Submission Control No: 269876. https://www.astrazeneca.ca/content/dam/az-ca/downloads/productinformation/imfinzi-product-monograph-en.pdf.

[61] Antonia S.J., López-Martin J.A., Bendell J., Ott P.A., Taylor M., Eder J.P., Jäger D., Pietanza M.C., Le D.T., de Braud F. (2016). Nivolumab alone and nivolumab plus ipilimumab in recurrent small-cell lung cancer (CheckMate 032): A multicentre, open-label, phase 1/2 trial. Lancet Oncol..

[62] Leal T., Wang Y., Dowlati A., Lewis D.A., Chen Y., Mohindra A.R., Razaq M., Ahuja H.G., Liu J., King D.M. (2020). Randomized phase II clinical trial of cisplatin/carboplatin and etoposide (CE) alone or in combination with nivolumab as frontline therapy for extensive-stage small-cell lung cancer (ES-SCLC): ECOG-ACRIN EA5161. J. Clin. Oncol..

[63] Owonikoko T.K., Park K., Govindan R., Ready N., Reck M., Peters S., Dakhil S.R., Navarro A., Rodríguez-Cid J., Schenker M. (2021). Nivolumab and Ipilimumab as Maintenance Therapy in Extensive-Disease Small-Cell Lung Cancer: CheckMate 451. J. Clin. Oncol..

[64] Cheng Y., Han L., Wu L., Chen J., Sun H., Wen G., Ji Y., Dvorkin M., Shi J., Pan Z. (2022). Effect of First-Line Serplulimab vs. Placebo Added to Chemotherapy on Survival in Patients with Extensive-Stage Small-cell Lung Cancer: The ASTRUM-005 Randomized Clinical Trial. JAMA.

[65] Wang J., Zhou C., Yao W., Wang Q., Min X., Chen G., Xu X., Li X., Xu F., Fang Y. (2022). Adebrelimab or placebo plus carboplatin and etoposide as first-line treatment for extensive-stage small-cell lung cancer (CAPSTONE-1): A multicentre, randomised, double-blind, placebo-controlled, phase 3 trial. Lancet Oncol..

[66] Targeted Oncology FDA Grants Orphan Drug Status to Serplulimab for Small-cell Lung Cancer. https://www.targetedonc.com/view/fda-grants-orphan-drug-status-to-serplulimab-for-small-cell-lung-cancer.

[67] Owonikoko T.K., Behera M., Chen Z., Bhimani C., Curran W.J., Khuri F.R., Ramalingam S.S. (2012). A systematic analysis of efficacy of second-line chemotherapy in sensitive and refractory small-cell lung cancer. J. Thorac. Oncol..

[68] Baize N., Monnet I., Greillier L., Geier M., Lena H., Janicot H., Vergnenegre A., Crequit J., Lamy R., Auliac J.B. (2020). Carboplatin plus etoposide versus topotecan as second-line treatment for patients with sensitive relapsed small-cell lung cancer: An open-label, multicentre, randomised, phase 3 trial. Lancet Oncol..

[69] O’Sullivan D.E., Cheung W.Y., Syed I.A., Moldaver D., Shanahan M.K., Bebb D.G., Sit C., Brenner D.R., Boyne D.J. (2021). Real-World Treatment Patterns, Clinical Outcomes, and Health Care Resource Utilization in Extensive-Stage Small-cell Lung Cancer in Canada. Curr. Oncol..

[70] Cramer-van der Welle C.M., Schramel F.M.N.H., van Leeuwen A.S., Groen H.J.M., van de Garde E.M.W. (2020). Real-world treatment patterns and outcomes of patients with extensive disease small-cell lung cancer. Eur. J. Cancer Care.

[71] Tendler S., Zhan Y., Pettersson A., Lewensohn R., Viktorsson K., Fang F., De Petris L. (2020). Treatment patterns and survival outcomes for small-cell lung cancer patients—A Swedish single center cohort study. Acta Oncol..

[72] Steffens C.C., Elender C., Hutzschenreuter U., Dille S., Binninger A., Spring L., Jänicke M., Marschner N. (2019). Treatment and outcome of 432 patients with extensive-stage small-cell lung cancer in first, second and third line-Results from the prospective German TLK cohort study. Lung Cancer.

[73] (2021). Topotecan Hydrochloride for Injection Product Monograph. Pfizer Canada ULC. Submission Control No.: 245735.. https://pdf.hres.ca/dpd_pm/00060717.pdf.

[74] von Pawel J., Schiller J.H., Shepherd F.A., Fields S.Z., Kleisbauer J.P., Chrysson N.G., Stewart D.J., Clark P.I., Palmer M.C., Depierre A. (1999). Topotecan versus cyclophosphamide, doxorubicin, and vincristine for the treatment of recurrent small-cell lung cancer. J. Clin. Oncol..

[75] Edelman M.J., Dvorkin M., Laktionov K., Navarro A., Juan-Vidal O., Kozlov V., Golden G., Jordan O., Deng C.Q., Bentsion D. (2022). Randomized phase 3 study of the anti-disialoganglioside antibody dinutuximab and irinotecan vs. irinotecan or topotecan for second-line treatment of small-cell lung cancer. Lung Cancer.

[76] (2022). Zepzelca (Lurbinectedin) Product Monograph. Jazz Pharmaceuticals Canada Incorporated. Submission Control No.: 259506. https://www.zepzelca.ca/sites/default/files/pdf/zepzelca.ca.PM-en.pdf.

[77] Canadian Agency for Drugs and Technologies in Health CADTH Reimbursement Recommendation: Lurbinectedin (Zepzelca). https://www.cadth.ca/sites/default/files/DRR/2023/PC0281%20Zepzelca%20-%20Confidental%20Final%20CADTH%20Recommendation%20(Redacted)-KH_SC%20-%20KH-meta.pdf.

[78] Paz-Ares L., Ciuleanu T., Navarro A., Fulop A., Cousin S., Bonanno L., Smit E., Chiappori A., Olmedo M.E., Horvath I. (2021). PL02.03 Lurbinectedin/Doxorubicin versus CAV or Topotecan in Relapsed SCLC Patients: Phase III Randomized ATLANTIS Trial. J. Thorac. Oncol..

[79] Masters G.A., Declerck L., Blanke C., Sandler A., DeVore R., Miller K., Johnson D. (2003). Phase II trial of gemcitabine in refractory or relapsed small-cell lung cancer: Eastern Cooperative Oncology Group Trial 1597. J. Clin. Oncol..

[80] Hoang T., Kim K., Jaslowski A., Koch P., Beatty P., McGovern J., Quisumbing M., Shapiro G., Witte R., Schiller J.H. (2003). Phase II study of second-line gemcitabine in sensitive or refractory small-cell lung cancer. Lung Cancer.

[81] Pietanza M.C., Kadota K., Huberman K., Sima C.S., Fiore J.J., Sumner D.K., Travis W.D., Heguy A., Ginsberg M.S., Holodny A.I. (2012). Phase II trial of temozolomide in patients with relapsed sensitive or refractory small-cell lung cancer, with assessment of methylguanine-DNA methyltransferase as a potential biomarker. Clin. Cancer Res..

[82] Furuse K., Kubota K., Kawahara M., Takada M., Kimura I., Fujii M., Ohta M., Hasegawa K., Yoshida K., Nakajima S. (1996). Phase II study of vinorelbine in heavily previously treated small-cell lung cancer. Japan Lung Cancer Vinorelbine Study Group. Oncology.

[83] Goldman J.W., Cummings A.L., Mendenhall M.A., Velez M.A., Babu S., Johnson T.T., Alcantar J.M., Dakhil S.R., Kanamori D.E., Lawler W.E. (2022). Primary analysis from the phase 2 study of continuous talazoparib (TALA) plus intermittent low-dose temozolomide (TMZ) in patients with relapsed or refractory extensive-stage small-cell lung cancer (ES-SCLC). ASCO 2022. J. Clin. Oncol..

[84] Farago A.F., Yeap B.Y., Stanzione M., Hung Y.P., Heist R.S., Marcoux J.P., Zhong J., Rangachari D., Barbie D.A., Phat S. (2019). Combination Olaparib and Temozolomide in Relapsed Small-Cell Lung Cancer. Cancer Discov..

[85] Pietanza M.C., Waqar S.N., Krug L.M., Dowlati A., Hann C.L., Chiappori A., Owonikoko T.K., Woo K.M., Cardnell R.J., Fujimoto J. (2018). Randomized, Double-Blind, Phase II Study of Temozolomide in Combination with Either Veliparib or Placebo in Patients with Relapsed-Sensitive or Refractory Small-Cell Lung Cancer. J. Clin. Oncol..

[86] Kim J.W., Hafez N., Soliman H.H., Fu S., Kato S., Lara P., Vaishampayan U.N., Razak A.R.A., Cardin D.B., Munster P.N. (2020). Preliminary efficacy data of platinum-pretreated small-cell lung cancer (SCLC) cohort of NCI 9881 study: A phase II study of cediranib in combination with olaparib in advanced solid tumors. J. Clin. Oncol..

[87] Ma S., He Z., Wang L., Wu Y., Yang S., Chen H., Wu Y., Mo Y., Liu Y., Wang Q. (2022). Sintilimab plus anlotinib as second or further-line therapy for small-cell lung cancer: An objective performance trial. J. Clin. Oncol..

[88] Liu Q., Xu J.Y., Xu Y.H., Chen M., Deng L.C., Wu J.P., Zhou T., Zhang L.Q., Tan J., Pu X.X. (2022). Efficacy and safety of apatinib as second or later-line therapy in extensive-stage small-cell lung cancer: A prospective, exploratory, single-arm, multi-center clinical trial. Transl. Lung Cancer Res..

[89] AbbVie News Center (2019). AbbVie Discontinues Rovalpituzumab Tesirine (Rova-T) Research and Development Program. Press Release. https://news.abbvie.com/news/press-releases/abbvie-discontinues-rovalpituzumab-tesirine-rova-t-research-and-development-program.htm.

[90] Hipp S., Voynov V., Drobits-Handl B., Giragossian C., Trapani F., Nixon A.E., Scheer J.M., Adam P.J. (2020). A Bispecific DLL3/CD3 IgG-Like T-Cell Engaging Antibody Induces Antitumor Responses in Small-cell Lung Cancer. Clin. Cancer Res..

[91] Wermke M., Felip E., Gambardella V., Kuboki Y., Morgensztern D., Hamed Z.O., Liu M., Studeny M., Owonikoko T.K. (2022). Phase I trial of the DLL3/CD3 bispecific T-cell engager BI 764532 in DLL3-positive small-cell lung cancer and neuroendocrine carcinomas. Future Oncol..

[92] Jeremic B., Shibamoto Y., Nikolic N., Milicic B., Milisavljevic S., Dagovic A., Aleksandrovic J., Radosavljevic-Asic G. (1999). Role of radiation therapy in the combined-modality treatment of patients with extensive disease small-cell lung cancer: A randomized study. J. Clin. Oncol..

[93] Slotman B.J., van Tinteren H., Praag J.O., Knegjens J.L., El Sharouni S.Y., Hatton M., Keijser A., Faivre-Finn C., Senan S. (2015). Use of thoracic radiotherapy for extensive stage small-cell lung cancer: A phase 3 randomised controlled trial. Lancet.

[94] Gore E.M., Hu C., Sun A.Y., Grimm D.F., Ramalingam S.S., Dunlap N.E., Higgins K.A., Werner-Wasik M., Allen A.M., Iyengar P. (2017). Randomized Phase II Study Comparing Prophylactic Cranial Irradiation Alone to Prophylactic Cranial Irradiation and Consolidative Extracranial Irradiation for Extensive-Disease Small-cell Lung Cancer (ED SCLC): NRG Oncology RTOG 0937. J. Thorac. Oncol..

[95] Palma D.A., Warner A., Louie A.V., Senan S., Slotman B., Rodrigues G.B. (2016). Thoracic Radiotherapy for Extensive Stage Small-Cell Lung Cancer: A Meta-Analysis. Clin. Lung Cancer.

[96] Rathod S., Jeremic B., Dubey A., Giuliani M., Bashir B., Chowdhury A., Liang Y., Pereira S., Agarwal J., Koul R. (2019). Role of thoracic consolidation radiation in extensive stage small-cell lung cancer: A systematic review and meta-analysis of randomised controlled trials. Eur. J. Cancer.

[97] Slotman B.J., van Tinteren H., Praag J.O., Knegjens J.L., El Sharouni S.Y., Hatton M., Keijser A., Faivre-Finn C., Senan S. (2015). Radiotherapy for extensive stage small-cell lung cancer—Authors’ reply. Lancet.

[98] Reinmuth N., Garassino M.C., Trukhin D., Hochmair M.J., Özgüroglu M., Havel L., Goldman J., Chen Y., Losonczy G., Spinnato F. (2021). P48.03 First-Line Durvalumab plus Platinum-Etoposide in ES-SCLC: Exploratory Analyses Based on Extent of Disease in CASPIAN. J. Thorac. Oncol..

[99] Sun A., Abdulkarim B., Blais N., Greenland J., Louie A.V., Melosky B., Schellenberg D., Snow S., Liu G. (2023). Use of radiation therapy among patients with Extensive-stage Small-cell lung cancer receiving Immunotherapy: Canadian consensus recommendations. Lung Cancer.

[100] Ge W., Xu H., Yan Y., Cao D. (2018). The effects of prophylactic cranial irradiation versus control on survival of patients with extensive-stage small-cell lung cancer: A meta-analysis of 14 trials. Radiat. Oncol..

[101] Rule W.G., Foster N.R., Meyers J.P., Ashman J.B., Vora S.A., Kozelsky T.F., Garces Y.I., Urbanic J.J., Salama J.K., Schild S.E. (2015). Prophylactic cranial irradiation in elderly patients with small-cell lung cancer: Findings from a North Central Cancer Treatment Group pooled analysis. J. Geriatr. Oncol..

[102] Schild S.E., Foster N.R., Meyers J.P., Ross H.J., Stella P.J., Garces Y.I., Olivier K.R., Molina J.R., Past L.R., Adjei A.A. (2012). Prophylactic cranial irradiation in small-cell lung cancer: Findings from a North Central Cancer Treatment Group Pooled Analysis. Ann. Oncol..

[103] Viani G.A., Boin A.C., Ikeda V.Y., Vianna B.S., Silva R.S., Santanella F. (2012). Thirty years of prophylactic cranial irradiation in patients with small-cell lung cancer: A meta-analysis of randomized clinical trials. J. Bras. Pneumol..

[104] Slotman B., Faivre-Finn C., Kramer G., Rankin E., Snee M., Hatton M., Postmus P., Collette L., Musat E., Senan S. (2007). Prophylactic cranial irradiation in extensive small-cell lung cancer. N. Engl. J. Med..

[105] Takahashi T., Yamanaka T., Seto T., Harada H., Nokihara H., Saka H., Nishio M., Kaneda H., Takayama K., Ishimoto O. (2017). Prophylactic cranial irradiation versus observation in patients with extensive-disease small-cell lung cancer: A multicentre, randomised, open-label, phase 3 trial. Lancet Oncol..

[106] Simone C.B., Bogart J.A., Cabrera A.R., Daly M.E., DeNunzio N.J., Detterbeck F., Faivre-Finn C., Gatschet N., Gore E., Jabbour S.K. (2020). Radiation Therapy for Small-cell Lung Cancer: An ASTRO Clinical Practice Guideline. Pract. Radiat. Oncol..

[107] Wolfson A.H., Bae K., Komaki R., Meyers C., Movsas B., Le Pechoux C., Werner-Wasik M., Videtic G.M., Garces Y.I., Choy H. (2011). Primary analysis of a phase II randomized trial Radiation Therapy Oncology Group (RTOG) 0212: Impact of different total doses and schedules of prophylactic cranial irradiation on chronic neurotoxicity and quality of life for patients with limited-disease small-cell lung cancer. Int. J. Radiat. Oncol. Biol. Phys..

[108] Le Péchoux C., Dunant A., Senan S., Wolfson A., Quoix E., Faivre-Finn C., Ciuleanu T., Arriagada R., Jones R., Wanders R. (2009). Standard-dose versus higher-dose prophylactic cranial irradiation (PCI) in patients with limited-stage small-cell lung cancer in complete remission after chemotherapy and thoracic radiotherapy (PCI 99-01, EORTC 22003-08004, RTOG 0212, and IFCT 99-01): A randomised clinical trial. Lancet Oncol..

[109] Rodríguez de Dios N., Couñago F., Murcia-Mejía M., Rico-Oses M., Calvo-Crespo P., Samper P., Vallejo C., Luna J., Trueba I., Sotoca A. (2021). Randomized Phase III Trial of Prophylactic Cranial Irradiation with or without Hippocampal Avoidance for Small-Cell Lung Cancer (PREMER): A GICOR-GOECP-SEOR Study. J. Clin. Oncol..

[110] Belderbos J.S.A., De Ruysscher D.K.M., De Jaeger K., Koppe F., Lambrecht M.L.F., Lievens Y.N., Dieleman E.M.T., Jaspers J.P.M., Van Meerbeeck J.P., Ubbels F. (2021). Phase 3 Randomized Trial of Prophylactic Cranial Irradiation with or without Hippocampus Avoidance in SCLC (NCT01780675). J. Thorac. Oncol..

[111] Rittberg R., Banerji S., Kim J.O., Rathod S., Dawe D.E. (2021). Treatment and Prevention of Brain Metastases in Small-cell Lung Cancer. Am. J. Clin. Oncol..

[112] Hirsch F.R., Paulson O.B., Hansen H.H., Larsen S.O. (1983). Intracranial metastases in small-cell carcinoma of the lung. Prognostic aspects. Cancer.

[113] Seute T., Leffers P., ten Velde G.P., Twijnstra A. (2004). Neurologic disorders in 432 consecutive patients with small-cell lung carcinoma. Cancer.

[114] Kristjansen P.E., Kristensen C.A. (1993). The role of prophylactic cranial irradiation in the management of small-cell lung cancer. Cancer Treat. Rev..

[115] Kepka L., Socha J., Sas-Korczynska B. (2021). Radiotherapy for brain metastases from small-cell lung cancer in distinct clinical indications and scenarios. J. Thorac. Dis..

[116] Grossi F., Scolaro T., Tixi L., Loprevite M., Ardizzoni A. (2001). The role of systemic chemotherapy in the treatment of brain metastases from small-cell lung cancer. Crit. Rev. Oncol. Hematol..

[117] Seute T., Leffers P., Wilmink J.T., ten Velde G.P., Twijnstra A. (2006). Response of asymptomatic brain metastases from small-cell lung cancer to systemic first-line chemotherapy. J. Clin. Oncol..

[118] van Grinsven E.E., Nagtegaal S.H.J., Verhoeff J.J.C., van Zandvoort M.J.E. (2021). The Impact of Stereotactic or Whole Brain Radiotherapy on Neurocognitive Functioning in Adult Patients with Brain Metastases: A Systematic Review and Meta-Analysis. Oncol. Res. Treat..

[119] Brown P.D., Pugh S., Laack N.N., Wefel J.S., Khuntia D., Meyers C., Choucair A., Fox S., Suh J.H., Roberge D. (2013). Memantine for the prevention of cognitive dysfunction in patients receiving whole-brain radiotherapy: A randomized, double-blind, placebo-controlled trial. Neuro Oncol..

[120] Brown P.D., Gondi V., Pugh S., Tome W.A., Wefel J.S., Armstrong T.S., Bovi J.A., Robinson C., Konski A., Khuntia D. (2020). Hippocampal Avoidance During Whole-Brain Radiotherapy Plus Memantine for Patients with Brain Metastases: Phase III Trial NRG Oncology CC001. J. Clin. Oncol..

[121] Vogelbaum M.A., Brown P.D., Messersmith H., Brastianos P.K., Burri S., Cahill D., Dunn I.F., Gaspar L.E., Gatson N.T.N., Gondi V. (2022). Treatment for Brain Metastases: ASCO-SNO-ASTRO Guideline. J. Clin. Oncol..

[122] Wegner R.E., Olson A.C., Kondziolka D., Niranjan A., Lundsford L.D., Flickinger J.C. (2011). Stereotactic radiosurgery for patients with brain metastases from small-cell lung cancer. Int. J. Radiat. Oncol. Biol. Phys..

[123] Robin T.P., Jones B.L., Amini A., Koshy M., Gaspar L.E., Liu A.K., Nath S.K., Kavanagh B.D., Camidge D.R., Rusthoven C.G. (2018). Radiosurgery alone is associated with favorable outcomes for brain metastases from small-cell lung cancer. Lung Cancer.

[124] Rusthoven C.G., Yamamoto M., Bernhardt D., Smith D.E., Gao D., Serizawa T., Yomo S., Aiyama H., Higuchi Y., Shuto T. (2020). Evaluation of First-line Radiosurgery vs. Whole-Brain Radiotherapy for Small-cell Lung Cancer Brain Metastases: The FIRE-SCLC Cohort Study. JAMA Oncol..

[125] Gaebe K., Li A.Y., Park A., Parmar A., Lok B.H., Sahgal A., Chan K.K.W., Erickson A.W., Das S. (2022). Stereotactic radiosurgery versus whole brain radiotherapy in patients with intracranial metastatic disease and small-cell lung cancer: A systematic review and meta-analysis. Lancet Oncol..

[126] Huang Y.H., Klingbeil O., He X.Y., Wu X.S., Arun G., Lu B., Somerville T.D.D., Milazzo J.P., Wilkinson J.E., Demerdash O.E. (2018). POU2F3 is a master regulator of a tuft cell-like variant of small-cell lung cancer. Genes. Dev..

[127] Rudin C.M., Poirier J.T., Byers L.A., Dive C., Dowlati A., George J., Heymach J.V., Johnson J.E., Lehman J.M., MacPherson D. (2019). Molecular subtypes of small-cell lung cancer: A synthesis of human and mouse model data. Nat. Rev. Cancer.

[128] Gay C.M., Stewart C.A., Park E.M., Diao L., Groves S.M., Heeke S., Nabet B.Y., Fujimoto J., Solis L.M., Lu W. (2021). Patterns of transcription factor programs and immune pathway activation define four major subtypes of SCLC with distinct therapeutic vulnerabilities. Cancer Cell.

[129] McColl K., Wildey G., Sakre N., Lipka M.B., Behtaj M., Kresak A., Chen Y., Yang M., Velcheti V., Fu P. (2017). Reciprocal expression of INSM1 and YAP1 defines subgroups in small-cell lung cancer. Oncotarget.

[130] Skoulidis F., Byers L.A., Diao L., Papadimitrakopoulou V.A., Tong P., Izzo J., Behrens C., Kadara H., Parra E.R., Canales J.R. (2015). Co-occurring genomic alterations define major subsets of KRAS-mutant lung adenocarcinoma with distinct biology, immune profiles, and therapeutic vulnerabilities. Cancer Discov..

[131] George J., Lim J.S., Jang S.J., Cun Y., Ozretić L., Kong G., Leenders F., Lu X., Fernández-Cuesta L., Bosco G. (2015). Comprehensive genomic profiles of small-cell lung cancer. Nature.

[132] Baine M.K., Hsieh M.S., Lai W.V., Egger J.V., Jungbluth A.A., Daneshbod Y., Beras A., Spencer R., Lopardo J., Bodd F. (2020). SCLC Subtypes Defined by ASCL1, NEUROD1, POU2F3, and YAP1: A Comprehensive Immunohistochemical and Histopathologic Characterization. J. Thorac. Oncol..

[133] Megyesfalvi Z., Barany N., Lantos A., Valko Z., Pipek O., Lang C., Schwendenwein A., Oberndorfer F., Paku S., Ferencz B. (2022). Expression patterns and prognostic relevance of subtype-specific transcription factors in surgically resected small-cell lung cancer: An international multicenter study. J. Pathol..

[134] Dora D., Rivard C., Yu H., Pickard S.L., Laszlo V., Harko T., Megyesfalvi Z., Gerdan C., Dinya E., Hoetzenecker K. (2023). Protein Expression of immune checkpoints STING and MHCII in small-cell lung cancer. Cancer Immunol. Immunother..

[135] Moore S.M., Zhan L.J., Liu G., Rittberg R., Patel D., Chowdhury D., Leung B., Cheng S., Mckinnon M., Khan K. (2022). EP03.01-016 The Canadian Small-cell Lung Cancer Database (CASCADE): Results from a Multi-Institutional Real-World Evidence Collaboration. J. Thorac. Oncol..

